# Quality and denoising in real‐time functional magnetic resonance imaging neurofeedback: A methods review

**DOI:** 10.1002/hbm.25010

**Published:** 2020-04-25

**Authors:** Stephan Heunis, Rolf Lamerichs, Svitlana Zinger, Cesar Caballero‐Gaudes, Jacobus F. A. Jansen, Bert Aldenkamp, Marcel Breeuwer

**Affiliations:** ^1^ Department of Electrical Engineering Eindhoven University of Technology Eindhoven The Netherlands; ^2^ Department of Research and Development Epilepsy Centre Kempenhaeghe Heeze The Netherlands; ^3^ Philips Research Eindhoven The Netherlands; ^4^ Basque Center on Cognition Brain and Language San Sebastian Spain; ^5^ Department of Radiology Maastricht University Medical Centre Maastricht The Netherlands; ^6^ School for Mental Health and Neuroscience Maastricht The Netherlands; ^7^ Laboratory for Clinical and Experimental Neurophysiology, Neurobiology and Neuropsychology Ghent University Hospital Ghent Belgium; ^8^ Department of Neurology Maastricht University Medical Center Maastricht The Netherlands; ^9^ Department of Biomedical Engineering Eindhoven University of Technology Eindhoven The Netherlands; ^10^ Philips Healthcare Best The Netherlands

**Keywords:** denoising, fMRI, neurofeedback, quality, real‐time, reproducibility

## Abstract

Neurofeedback training using real‐time functional magnetic resonance imaging (rtfMRI‐NF) allows subjects voluntary control of localised and distributed brain activity. It has sparked increased interest as a promising non‐invasive treatment option in neuropsychiatric and neurocognitive disorders, although its efficacy and clinical significance are yet to be determined. In this work, we present the first extensive review of acquisition, processing and quality control methods available to improve the quality of the neurofeedback signal. Furthermore, we investigate the state of denoising and quality control practices in 128 recently published rtfMRI‐NF studies. We found: (a) that less than a third of the studies reported implementing standard real‐time fMRI denoising steps, (b) significant room for improvement with regards to methods reporting and (c) the need for methodological studies quantifying and comparing the contribution of denoising steps to the neurofeedback signal quality. Advances in rtfMRI‐NF research depend on reproducibility of methods and results. Notably, a systematic effort is needed to build up evidence that disentangles the various mechanisms influencing neurofeedback effects. To this end, we recommend that future rtfMRI‐NF studies: (a) report implementation of a set of standard real‐time fMRI denoising steps according to a proposed COBIDAS‐style checklist (https://osf.io/kjwhf/), (b) ensure the quality of the neurofeedback signal by calculating and reporting community‐informed quality metrics and applying offline control checks and (c) strive to adopt transparent principles in the form of methods and data sharing and support of open‐source rtfMRI‐NF software. Code and data for reproducibility, as well as an interactive environment to explore the study data, can be accessed at https://github.com/jsheunis/quality‐and‐denoising‐in‐rtfmri‐nf.

## 1 INTRODUCTION

*Real‐time fMRI*: Real‐time functional magnetic resonance imaging (rtfMRI) involves the dynamic processing, analysis and visualisation of a subject's changing blood oxygen level‐dependent (BOLD) signal and related information while the subject is inside the MRI scanner. It was initially proposed and developed by Cox, Jesmanowicz, and Hyde (1995) as a tool for real‐time data quality monitoring, functional activation mapping and interactive experimental design. Since its inception this technology has expanded to include a variety of software tools that allow pre‐experimental and pre‐surgical functional localisation (Binder, 2011; Hirsch et al., 2000), real‐time functional activity mapping (as is available in the software accompanying MRI systems from all major vendors), brain computer interfacing (e.g. Sorger, Reithler, Dahmen, & Goebel, 2012), brain state decoding (LaConte, 2011), real‐time neurofeedback (Sitaram et al., 2017) and interactive demonstrations for educational purposes (Weiskopf et al., 2007).

*BOLD self‐regulation through neurofeedback*: Neurofeedback training as an application of real‐time fMRI (rtfMRI‐NF) has gained much interest in the past decade due to its ability to help subjects achieve learned regulation of regional brain activation, as was initially demonstrated by Yoo and Jolesz (2002) in a motor task experiment. Interested readers are referred to Sitaram et al. (2017) for a recent review of rtfMRI‐NF functionality, technology and applications. Shortly, by feeding a representation of quantified brain activity back to the subject in the scanner in near‐real‐time, and asking subjects to increase or decrease the presented metric by adopting one of several possible training strategies (or none at all), subjects have been able to regulate their own BOLD signal. This is evidenced by increased activation levels and cluster sizes in the areas of interest measured over multiple training sessions (see, e.g. deCharms, 2007).

*Clinical applications*: In further steps, learned brain activity regulation through neurofeedback training has been used in neuropsychological and psychiatric disorders to test for behavioural correlates, aiming to investigate non‐invasive rtfMRI‐NF as an alternative to more invasive treatment modalities like pharmacological interventions, surgery or deep brain stimulation. Several studies have reported significantly beneficial behavioural, symptomatic or experiential changes after rtfMRI‐NF training in a variety of clinical or other populations, including major depressive disorder (Linden et al., 2012), tinnitus (Emmert et al., 2017), attention deficit and hyperactivity disorder (Alegria et al., 2017), obesity (Spetter et al., 2017) and nicotine cravings (Canterberry et al., 2013).

*Criticism and open questions*: In order for rtfMRI‐NF to show proven clinical utility and efficacy, reproducibility of methods, of results and of inferences are imperative^1^ (Goodman et al., 2016; Munafò et al., 2017). Evidence for widespread and clinically significant effects of rtfMRI‐NF training has however been called into question by recognising a lack of replication studies (Sulzer et al., 2013), of blinded placebo‐controlled study designs (Thibault, MacPherson, Lifshitz, Roth, & Raz, 2018) and of reproducible methods (Stoeckel et al., 2014). As an example, deCharms et al. (2005) showed in a pilot study that 8 out of 12 chronic pain patients (total *N =* 36 subjects) could learn to regulate the BOLD response in the rostral anterior cingulate cortex, leading to significant changes in pain perception in this group.

However, their subsequent study assessing adverse events associated with repeated fMRI scanning (Hawkinson et al., 2012) found no significant changes with regards to baseline in adverse event reporting in pain patients undergoing multiple rtfMRI neurofeedback sessions (69 out of total *N =* 114 patients). This apparent inability to replicate pilot findings in a larger sample size, which featured as a prominent discussion at the first Swiss rtfMRI Neurofeedback Conference (Decharms, 2012), suggests the need to re‐evaluate current small‐sample positive findings and incentivise the publication of null results, so as to counteract publication bias in neurofeedback literature.

Ongoing debate in the field still focuses on important and unanswered questions and challenges, many previously highlighted by Sulzer, Haller, et al. (2013). For example: How is neurofeedback learning and its success quantified, and is this quantification consistent enough to allow generalisation across studies? How do outcomes of active neurofeedback training perform compared to that of alternative and conventional treatment methods, and compared to outcomes of sham neurofeedback? Are perceived clinical benefits specific to certain populations, individual learning strategies, feedback calculation, feedback display, study design, data analysis, or other sources of variance? Widespread evidence to support specific, robust and reproducible findings for these research questions is still lacking, which should be seen as an incentive to improve methods reproducibility and to conduct large‐scale replication studies investigating specific effects of rtfMRI‐NF.

*Methods reproducibility and quality*: Central to several aspects influencing the reproducibility of both methods and results in rtfMRI‐NF is the concept of quality, which pertains to real‐time fMRI data, to the neurofeedback signal and to methods reporting. Take the assumption that the neurofeedback signal calculated from the real‐time fMRI data aims to represent brain activity relating to the subject's ongoing cognitive processes (Koush, Zvyagintsev, Dyck, Mathiak, & Mathiak, 2012). It is well‐known that the resting state or task‐induced BOLD signal contains several scanner‐, sequence‐, subject‐ or experiment‐related nuisance signals and artefacts (Caballero‐Gaudes & Reynolds, 2017; Liu, 2016; Murphy, Birn, & Bandettini, 2013; Power et al., 2014). If such confounding factors are not sufficiently accounted for during acquisition or minimised through real‐time processing, the feedback signal will remain confounded and will thus not sufficiently reflect brain activity of interest. This may lead to sham learning or to a nuisance signal being trained instead of the subject's BOLD response (Koush et al., 2012; LaConte, 2011), which affects reproducibility of results and inferences. Similarly, doubts about the quality of the feedback signal can exist due to the as yet unknown influences of feedback presentation (e.g. the widely used thermometer display vs. a more naturalistic display or virtual environment) and feedback signal calculation (e.g. temporal smoothing parameters, signal scaling, and the way in which percentage signal change is calculated). Few studies in this field have meticulously investigated such detail. This, added to the lack of methods standardisation and best practices for methods reporting, hinders reproducibility and generalisability.

*Research goal*: The above‐mentioned open questions, methodological uncertainties and lack of standardisation should guide efforts to move towards improved reproducibility in the field of fMRI neurofeedback. Specifically, a systematic effort is needed to build up evidence that disentangles neurofeedback training outcomes from placebo effects, that clarifies the efficacy of neurofeedback compared to existing treatments, and that demonstrates the specificity of neurofeedback effects while accounting for other sources of variance.

To support this effort, this work reviews the methods currently available to the researcher to improve the data quality and signal‐to‐noise ratio (SNR) of the rtfMRI‐NF signal and of real‐time fMRI data and studies in general. Specifically, we investigate three research questions:

1. What are challenges to effective denoising and improving quality in rtfMRI‐NF?
2. Which steps have recent rtfMRI‐NF studies taken to improve data quality and SNR?
3. Which methods for denoising data and improving data quality and SNR are available to the researcher studying rtfMRI‐NF?

To preface addressing these questions, a background on the BOLD signal and its confounds and on the details of the calculated neurofeedback signal is provided. Although both acquisition and processing methods are covered in this work, focus is given to the latter. We conclude with a general discussion and future recommendations based on the reviewed literature.

## 2 BACKGROUND

### 2.1 The BOLD signal, noise, artefacts and correction methods

*The noisy BOLD signal*: The *T*_2_*‐weighted BOLD signal typically acquired using standard gradient‐echo echo‐planar imaging (EPI) in fMRI represents hemodynamic and metabolic responses, through a neurovascular coupling, to alterations in neuronal activity (Ogawa, Menon, Kim, & Ugurbil, 1998). It results from a complex interaction between neural metabolism, blood oxygen concentration (specifically the local concentration of paramagnetic deoxyhemoglobin), cerebral blood flow (CBF) and cerebral blood volume (CBV) (Logothetis, 2003).

Given its dependence on neuronal metabolism, cerebral blood flow/volume and the inherent properties of MRI (and the EPI sequence in particular), it should be no surprise that the BOLD signal has several confounds and remains difficult to isolate as a proxy for true neuronal activity (Diedrichsen & Shadmehr, 2005). fMRI is typically plagued by a variety of noise fluctuations and artefacts originating either from the subject, from the experimental conditions, from the inherent properties of the acquisition sequence, or from the scanner and its (interfering) environment.

*Denoising the BOLD signal*: Much research effort has been given to ridding fMRI of noise. These efforts can be divided into two main categories: *acquisition* and *data processing*. Acquisition methods typically entail pulse sequence alterations or MRI parameter choices that improve the BOLD sensitivity, increase SNR, or preempt and minimise the effects of artefacts that may occur during scanning. Data processing methods to remove noise have been widely reported and typically take the form of model‐based or model‐free methods. Examples of model‐based denoising or artefact removal steps in fMRI pre‐processing pipelines include: slice‐time correction, 3D volume realignment, frequency band filtering, spatial smoothing, distortion correction, outlier removal/scrubbing (Siegel et al., 2014), regression of movement parameter residuals (Friston, Williams, Howard, Frackowiak, & Turner, 1996), global signal regression (Power, Plitt, Laumann, & Martin, 2017) and physiological noise regression (Birn, Diamond, Smith, & Bandettini, 2006; Glover, Li, & Ress, 2000). Model‐free methods mainly include the identification and removal of artefacts through the use of spatial independent component analysis (ICA; Perlbarg et al., 2007; Griffanti et al., 2014). For a thorough understanding of fMRI noise and denoising methods, readers are referred to in‐depth reviews by Murphy et al. (2013), Power et al. (2014), Caballero‐Gaudes and Reynolds (2017), Liu (2016), Kundu et al. (2017) and Power et al. (2018).

*In the absence of noise correction*: Studies have investigated the implications of not correcting sufficiently for (or ignoring) fMRI noise, confounds and artefacts. Head motion, for example, has been shown to result in false activity patterns when coupled to the timing of the task paradigm (Hajnal et al., 1994), to cause simultaneous decreases in long‐distance correlations and increases in short‐distance correlations within functional connectivity networks (Power, Barnes, Snyder, Schlaggar, & Petersen, 2012), and to cause problems in interpretations of functional connectivity measures across groups (Van Dijk, Sabuncu, & Buckner, 2012). The hemodynamic response function (HRF) is known to vary spatially across the brain, as well as between subjects and between studies (Handwerker, Gonzalez‐Castillo, D'Esposito, & Bandettini, 2012; Huettel & McCarthy, 2001), but the time‐to‐peak in standard task‐fMRI experiments is typically assumed to be ~4–6 s brain‐wide. Gitelman, Penny, Ashburner, and Friston (2003) investigated this assumption and showed the importance of deconvolution prior to modelling psychophysiologic interactions when considering functional/effective connectivity measures across the brain. HRF variability was further explored in a recent study by Rangaprakash, Wu, Marinazzo, Hu, and Deshpande (2018) which found that, if not accounted for, it could lead to identification of false functional connectivity measures. Noise sources resulting in global signal fluctuations (e.g. respiratory cycles) can also lead to incorrect attribution of signal to brain activity if regional BOLD fluctuations are considered in isolation (Noll & Schneider, 1994), that is, without regard to possibly confounding global signal correlations.

Noise sources remain problematic whether fMRI data are considered in real‐time or offline. It is therefore important when considering real‐time fMRI to address these known noise fluctuations and artefacts so as to increase the BOLD SNR, and to consider the implications of not correcting for these nuisances.

### 2.2 Real‐time fMRI

**FIGURE 1:**
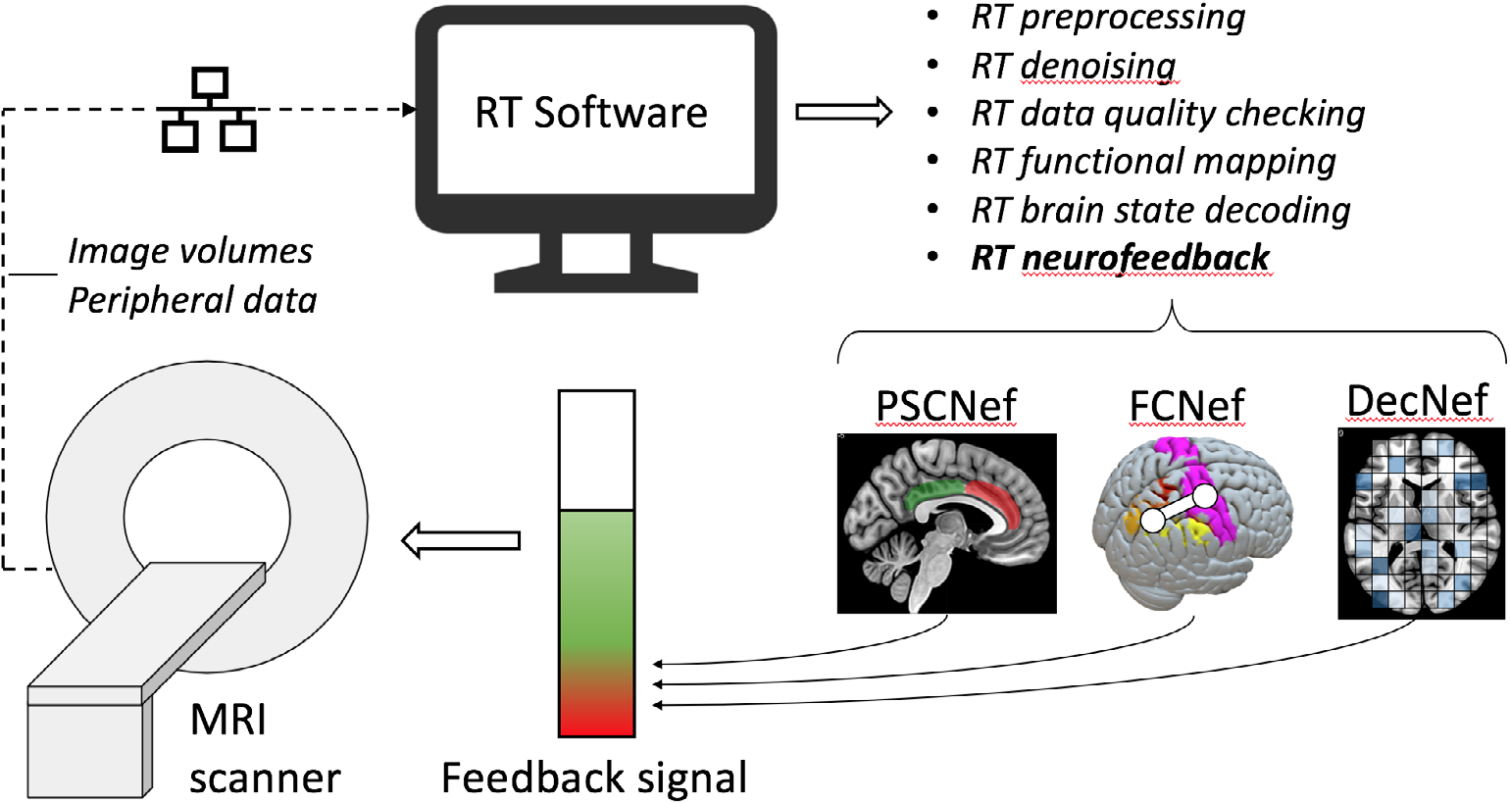
A typical real‐time fMRI technical setup, showing detailed components of a neurofeedback application. DecNef, decoded neurofeedback; FCNef, functional connectivity neurofeedback; RT, real‐time; PSCNef, percentage signal change neurofeedback

The vast majority of real‐time fMRI implementations use single echo echo‐planar imaging (EPI) as the preferred acquisition method, likely due to its prevalence in conventional functional imaging. Acquired slices are reconstructed on the MRI scanner hardware, and upon completion, each functional image volume is typically exported and shared on a local network from where it is accessible by the real‐time fMRI application software. Figure 1 illustrates a standard real‐time fMRI setup, including components of a neurofeedback application.

*Time frame definitions*: The ‘real‐time’ time frame is loosely defined to be the interval between two successive functional image scans, that is, the repetition time (TR), indicating that the concept ‘real‐time’ varies according to the application. Ideally, all required reconstruction, export and processing steps for each functional image should be completed sufficiently prior to or by the time the next image in the session is acquired, thus allowing the presentation of up‐to‐date *image* information to the researcher and/or subject. Given the nature of the HRF, real‐time fMRI not only includes a delay of one TR typically used for data processing (reported to be ~1–3 s in standard rtfMRI‐NF applications), but also a substantial delay due to the indirect measurement of neuronal activity (~4–6 s). As such, typical implementations of real‐time fMRI often only allow a representation of *brain activity* about 5 s or more after such changes occurred on a neuronal level, leading to the term ‘near‐real‐time’. This definition is distinct from the same term used by some studies to refer to a real‐time fMRI processing stream that delivers brain activity and other information within minutes after completing the functional scan session (e.g. Voyvodic, 1999). These time frame definitions assume a streamlined infrastructure for real‐time fMRI volume reconstruction and export with negligible latency issues, which in reality will vary and could result in potentially serious synchronisation challenges.

Note that neurofeedback presentation does not have to be synchronised with image acquisition and can be updated continuously or intermittently depending on the fMRI acquisition rate, software implementation and experimental design. Differences between continuous and intermittent feedback can also influence the selection of online pre‐processing and analysis strategies, as well as training goals and assumptions about the involved cognitive and neural processes. For such considerations, evidence from studies including Johnson et al. (2012), Oblak, Lewis‐Peacock, and Sulzer (2017), Emmert et al. (2017) and Hellrung et al. (2018) could be useful when selecting between feedback types.

*Real‐time processing steps*: The data processing steps necessary to derive a near‐real‐time representation of brain activity vary according to the application and implemented toolset, but typically follow the course of conventional task‐based or resting state fMRI analysis, where data are first pre‐processed to remove artefacts or noise fluctuations and then analysed with model‐based or model‐free statistical methods to extract information of interest. Real‐time fMRI neurofeedback pre‐processing typically consists of 3D volume realignment, spatial smoothing, linear or polynomial trend removal and temporal filtering, while few applications report the use of slice‐timing correction, physiological noise correction methods or real‐time distortion correction. These reported pre‐processing steps are delineated further in Section 4.

Univariate statistical analysis methods implemented in real‐time include recursive correlation between voxel time‐series and a reference vector (Cox et al., 1995), *t*‐tests (Voyvodic, 1999), multiple linear regression (Smyser, Grabowski, Frank, Haller, & Bolinger, 2001) and general linear model (GLM; Bagarinao, Matsuo, Nakai, & Sato, 2003). Multivariate methods applied to real‐time fMRI are less common, with the real‐time implementation of a support vector machine classifier (SVM; LaConte, Peltier, & Hu, 2007) being the first example, and sparse logistic regression (Shibata, Watanabe, Sasaki, & Kawato, 2011) and sparse multinomial or linear regression (Shibata, Watanabe, Kawato, & Sasaki, 2016) being used for recent real‐time pattern decoding.

**FIGURE 2:**
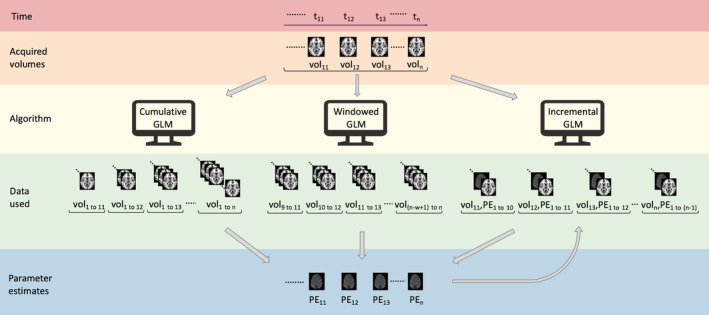
A representation of the three most commonly used real‐time general linear model (GLM) algorithms, indicating the differences in how data available for each iteration are incorporated into the algorithms. A cumulative GLM (cGLM) uses all available data at each iteration to calculate the parameter estimates per iteration. A windowed GLM (wGLM) uses a window size of the most recent *w* (= 3 in this example) volumes to calculate parameter estimates for a specific iteration. An incremental GLM (iGLM) incorporates volume data for each new iteration into an existing state. PE, parameter estimate; t, time; vol = volume

*Algorithmic adaptations*: To decrease the required per‐volume processing time, algorithms generally make use of sliding window (Gembris et al., 2000) or incremental approaches (Bagarinao et al., 2003) when analysing time‐series data (see Figure 2). While time‐windowed algorithms allow more sensitivity to temporal brain activity fluctuations by only analysing a recent subset of the acquired data, they are characterised by a decrease in statistical power (Weiskopf, Sitaram, et al., 2007), the converse being the case for incremental or cumulative algorithms that analyse all acquired data. A distinction is made here between incremental methods that use the data in each new iteration to update a growing statistical model so as to avoid recomputation (e.g. the incremental GLM developed by Bagarinao et al., 2003, that incrementally estimates and updates the coefficients of a GLM), and cumulative methods that repeat the operation during each iteration on all data acquired up to that iteration.

*Computational advances*: In general, real‐time fMRI pre‐processing and statistical analysis pipelines are simplified and/or optimised versions of their standard offline counterparts because priority is given to fast algorithms (those that converge in as few as possible iterations) and to the inclusion of the minimum sufficient steps to achieve an acceptable level of data quality, so as to decrease per‐volume processing time. This trade‐off between maintaining a high level of method accuracy and minimising the required per‐volume processing time has initially been a large constraint to expanding the complexity of real‐time fMRI processing steps, but has become increasingly easier to manage with advances in modern computing technology and algorithm development. The use of parallel computing using clusters (e.g. Bagarinao et al., 2003), multiple processing cores (e.g. Koush et al., 2017a), and parallel cloud computing (Wang et al., 2016; Cohen et al., 2017), as well as the use of graphical processing units (GPUs; Eklund, Andersson, & Knutsson, 2012; Scheinost et al., 2013; Misaki et al., 2015), allow substantial decreases in required per‐volume processing times and could accordingly afford real‐time fMRI tools a comparative level of complexity and accuracy as that of their offline counterparts. New research avenues become possible like whole‐brain real‐time fMRI (Misaki et al., 2015), full correlation matrix analysis (Wang et al., 2016), and complex processing for more effective noise removal (Misaki et al., 2015). With such computing power advancements, research outputs become more dependent on how the researcher selects MRI sequence parameters and signal processing steps, and less so on per‐volume time restrictions. This shift enables increases in real‐time BOLD quality.

### 2.3 Real‐time fMRI neurofeedback

The rtfMRI‐NF signal presented to the subject varies per study, but has been based on measures derived through three main computing methods: (a) BOLD activity percentage signal change typically in a single or differential region of interest (PSCNef), (b) functional connectivity between BOLD activity in multiple ROIs (FCNef) and (c) multivariate (or multivoxel) pattern analysis (MVPA), typically within a single ROI (DecNef).

**FIGURE 3:**
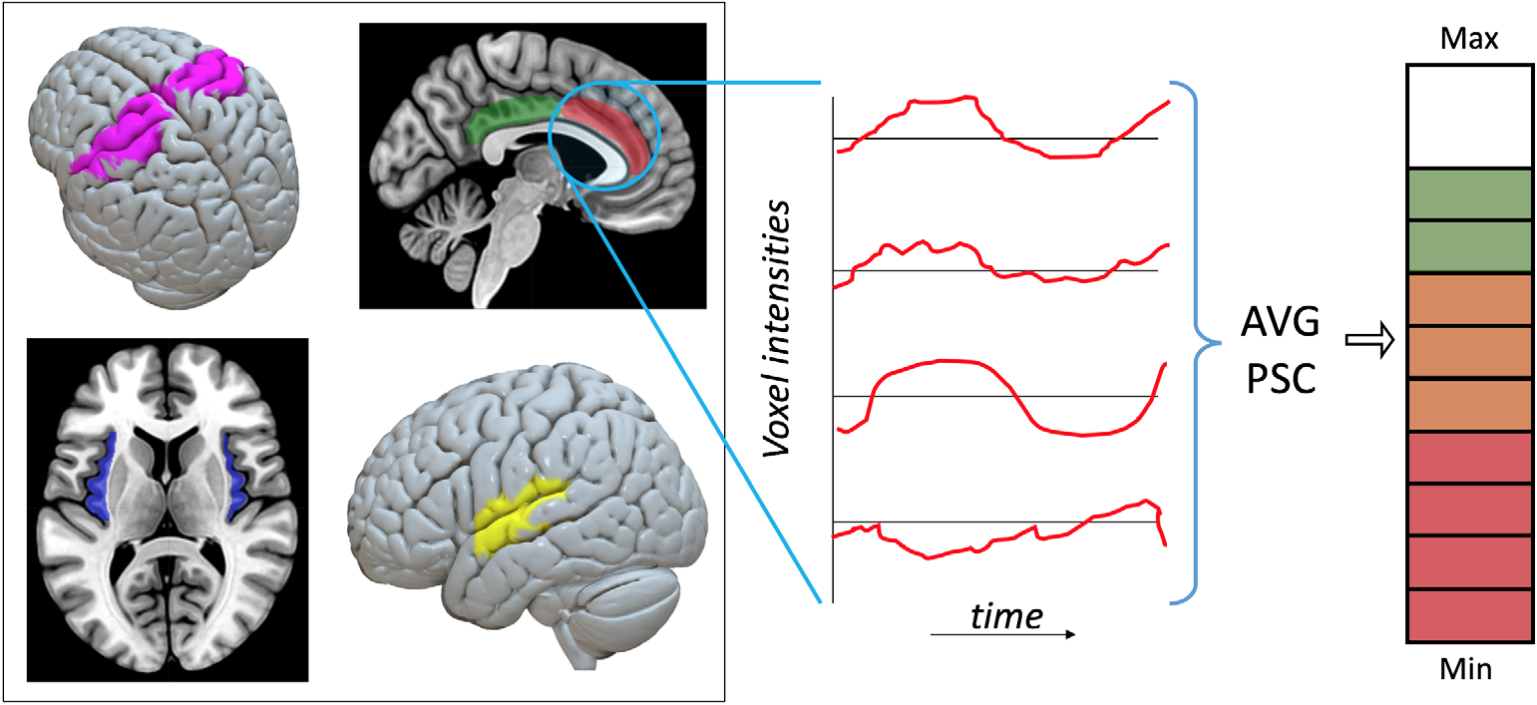
A linear neurofeedback signal (right) calculated as the average percentage signal change within the anterior cingulate cortex. Examples of other regions of interest are also displayed (left)

*Percentage signal change neurofeedback*: The majority of volunteer and patient rtfMRI‐NF studies have used a single or multiple ROI approach to calculate the feedback signal, specifically using the percentage signal change (PSCNef) of the spatially averaged signal obtained from all voxels within the defined ROI(s), as illustrated in Figure 3. Various regions of interest have been selected for different reasons, with the insula, amygdala, and the cingulate, auditory, visual and motor cortices often forming the basis for neurofeedback (Thibault et al., 2018). Regions of interest are most often acquired using a subject‐based functional localizer run before neurofeedback commences (Weiskopf, Sitaram, et al., 2007), although template based or anatomical ROIs have also been used. Several important factors need to be accounted for when using single ROIs as the feedback target. This includes increased signal dropout resulting from EPI imaging of lower or mid‐brain regions (e.g. the limbic system or medial temporal region) due to increased magnetic susceptibility gradients near air/tissue borders, leading to lower BOLD SNR.

**FIGURE 4:**
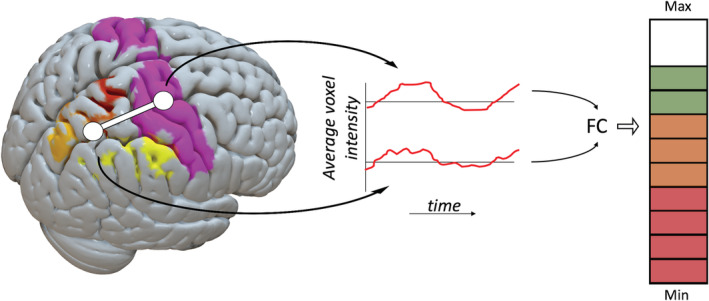
A linear neurofeedback signal (right) calculated as the functional connectivity (e.g. windowed Pearson's correlation) level between the motor and parietal cortex ROIs

*Functional connectivity neurofeedback*: FCNef (Watanabe, Sasaki, Shibata, & Kawato, 2017) was introduced to target applicable brain networks and their correlation rather than isolated activity in specific ROIs (Ruiz et al., 2013), and it has shown promise as an alternative to PSCNef. The principle is explained in Figure 4, where the average signal from different ROIs (in this case the motor and parietal cortices) is correlated across a moving time window to calculate the feedback signal. Various connectivity measures can be used as a basis for the neurofeedback signal, including Pearson's Correlation (Zilverstand, Sorger, Zimmermann, Kaas, & Goebel, 2014) and Dynamic Causal Modelling (Koush et al., 2013). When using FCNef, care has to be taken to prevent global signal fluctuations from biasing the calculated connectivity measure (and thus the feedback signal), based on concerns raised by Power et al. (2012) and Van Dijk et al. (2012) that were highlighted earlier.

**FIGURE 5:**
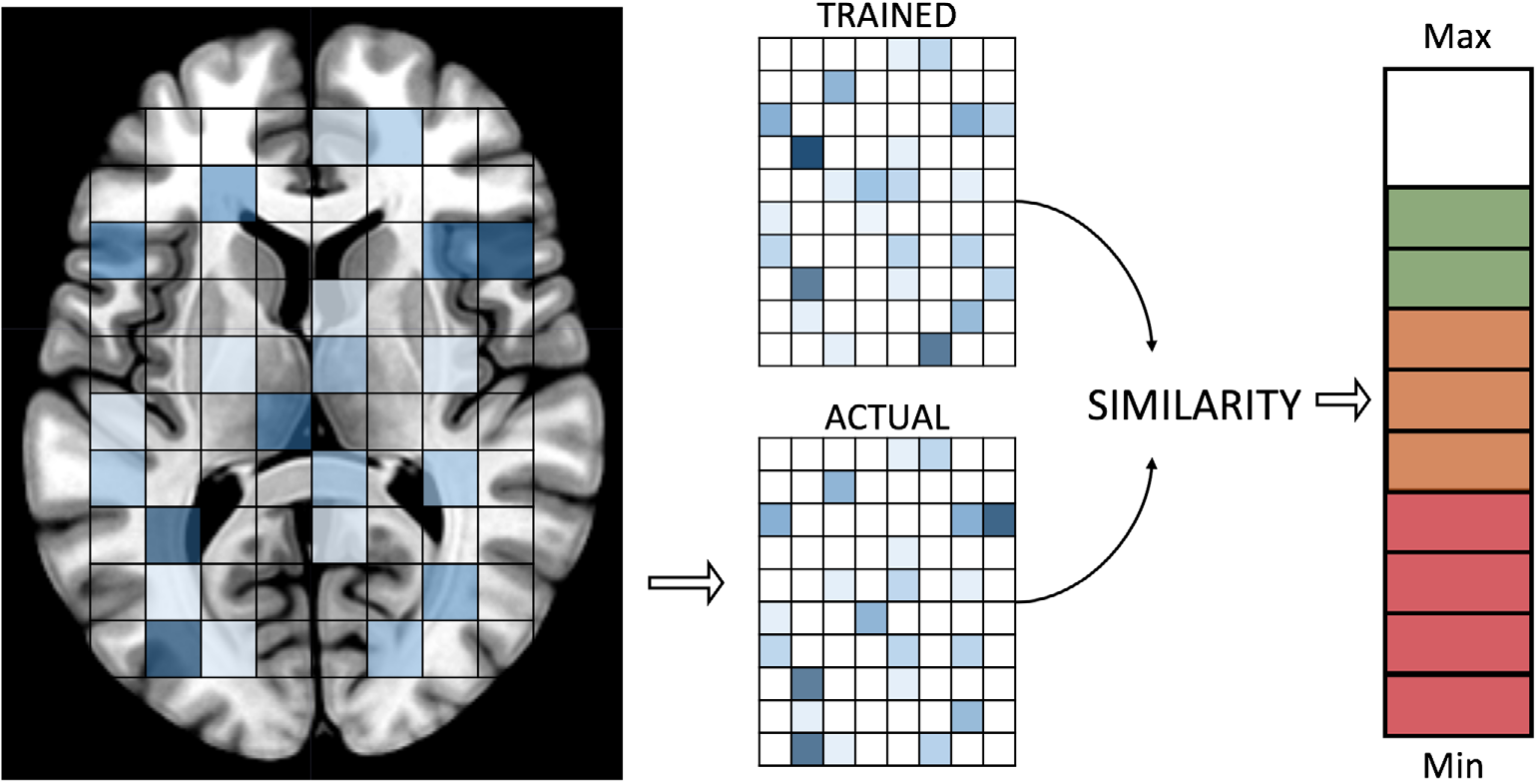
A linear neurofeedback signal (right) decoded as a representation of the similarity between voxel intensities in the trained pattern (resembling some known brain state) and the real‐time brain voxel pattern

*Decoded neurofeedback*: Real‐time fMRI multivoxel pattern analysis (also known as brain state decoding, decoded neurofeedback or DecNef, Watanabe et al., 2017) applies multivariate techniques to fMRI data, first by constructing a decoder using pre‐neurofeedback session data with known task‐modulation or states, which is then used in real‐time to decode each acquired volume for similarity to the target brain state pattern (see Figure 5). Support vector machine (SVM) algorithms for real‐time classification have been incorporated into several rtfMRI‐NF toolboxes (AFNI—LaConte et al., 2007; Turbo‐BrainVoyager—Sorger et al., 2010; FRIEND—Basilio et al., 2015). In addition, sparse logistic, sparse multinomial and sparse linear regression algorithms have been often used as decoders, depending on both the software implementation and the nature of the required neurofeedback signal (e.g. binary or linear). For further detail, LaConte (2011) and Watanabe et al. (2017) provide reviews of methodology and studies, respectively, using real‐time fMRI DecNef.

For more examples of studies using the methods above, readers are referred to Watanabe et al. (2017) and Thibault et al. (2018). Watanabe et al. (2017) explored advances in FCNef and DecNef based real‐time fMRI, providing a list of nine studies using these methods, explaining concepts and listing new challenges and possible solutions in the realm of FCNef and DecNef methods. Thibault et al. (2018) conducted a critical systematic review of 99 rtfMRI‐NF studies (mostly PSCNef) to evaluate the effectiveness of reported experimental protocols to train subjects to self‐regulate their BOLD signal.

Apart from the feedback type and target region, several aspects of rtfMRI‐NF can influence the ability of subjects to learn self‐regulation of the neurofeedback signal (Kadosh & Staunton, 2019). Experiments need to take account of the advantages or disadvantages of, amongst others, the regularity of neurofeedback presentation (continuous vs. intermittent), external rewards for learning outcomes, simultaneous visual display of task and feedback information, instructions given to subjects on learning strategy, variability in individual learning strategy of subjects, real‐time data quality measures, and the use of control groups and blinding in order to reach the full potential of a rigorously designed and reproducible rtfMRI‐NF experiment. Importantly, studies need to clarify these decisions (based on available evidence, pilot results or sound reasoning) and report their choices transparently, in aid of the effort to delineate the multiple mechanisms and influences leading to neurofeedback learning and accompanying behavioural effects.

## 3 QUALITY IN REAL‐TIME FMRI NEUROFEEDBACK

Quality is an umbrella term that is applicable to real‐time fMRI data, to the neurofeedback signal and to the methods reporting process. Generally, fMRI data quality is a measure of how well the acquired BOLD data reflects the signal of interest, that is, neural activity, and it is influenced by variability in multiple factors including the subject, the experimental design and acquisition (spatial resolution, image contrast, field strength etc.). If fMRI data quality is high, the implication is that signals that are *not* of interest (i.e. noise) are either absent from or not biasing our interpretation of the processed data, and there is lower possibility of making false inferences. This improves results and inferential reproducibility, and thus scientific progress. As an extension of fMRI data, high quality of the real‐time fMRI neurofeedback signal implies that a signal closely reflective of brain activity (and not of noise or artefacts) is fed back to the subject in real‐time. Quality in methods reporting implies that a published study contains enough information about the applicable experimental‐, acquisition‐ and data processing steps that would allow different researchers to reproduce the methods. Here, high quality has a direct and beneficial influence on methods reproducibility.

When aiming to improve quality in real‐time fMRI neurofeedback it is therefore advised to (a) separate the effects of noise (measurement‐, system‐, or subject‐based) from true BOLD fluctuations, (b) quantify and report the quality of real‐time fMRI data and the calculated neurofeedback signal and (c) accurately and sufficiently report the use of applicable real‐time fMRI denoising methods.

### 3.1 Measuring, comparing and reporting rtfMRI data quality

Traditionally, apart from expert visual inspection of fMRI datasets to identify low quality volumes/sessions/subjects/sites (as evidenced by visible artefacts in fMRI images like excessive motion, RF interference or ghosting), the temporal signal‐to‐noise ratio (tSNR) has been an important quantitative measure of fMRI data quality and the ability of an experiment to find effects of interest (Murphy, Bodurka, & Bandettini, 2007; Parrish, Gitelman, LaBar, & Mesulam, 2000; Welvaert & Rosseel, 2013). tSNR gives an indication of the per‐voxel signal fluctuations rated against the background noise fluctuations, with an example equation being:

(1) $$\text{tSNR}=\frac{\bar{S}}{\sigma }$$

Here, $\bar{S}$ and *σ* refer to the (per‐voxel) mean and *SD* of the fMRI time series, respectively. A variation of tSNR is the temporal contrast‐to‐noise ratio (tCNR, Geissler et al., 2007), which investigates the difference between functional contrast conditions (e.g. task activity vs. baseline activity) rather than considering signal fluctuations at all times. As such, tCNR could be defined as (Koush et al., 2012):

(2) $$\text{tCNR}=\frac{{\bar{S}}_{\text{contrast}}}{\sigma_{\mathrm{all}}}=\frac{{\bar{S}}_{\text{condition}}-{\bar{S}}_{\text{baseline}}}{\sqrt{\sigma^{2}\;}}=\left(\frac{\Delta S}{\bar{S}}*\text{tSNR}\right)$$

The part of Equation (2) in brackets provides a common definition of CNR (Krüger & Glover, 2001), where Δ*S* is the signal change due to an experimental condition. Equation (2) thus assumes that $\bar{S}$_condition_ = Δ*S*, and that $\bar{S}$_baseline_ = 0.

A simple fMRI quality inspection approach could be to compare the tSNR or tCNR values calculated before and after denoising to see if the change brings about a data quality increase. It should be noted that, depending on how noise and signal sources are defined spatially and on the type of condition and baseline choices, tSNR and tCNR values could vary and are not automatically standardised. Importantly, there is little consensus on an *SD* of tSNR and tCNR (Welvaert & Rosseel, 2013), which could hinder comparability between different sites and studies. Additionally, a single metric is unlikely to provide a full quantitative view of the quality of a complex signal such as fMRI, and further measures could be insightful.

*Quality tools and methods*: Historically, AFNI's real‐time fMRI module (Cox et al., 1995) supported the ability to display motion parameters to the subject in order the suppress head motion (Yang, Ross, Zhang, Stein, & Yang, 2005) and to feed back a display of variability in areas affected by physiological noise (e.g. ventricles) in order to reduce the *SD* of the fMRI signal (Bodurka, Gonzales‐Castillo, & Bandettini, 2009). These parameters can inherently also serve as real‐time quality indicators.

More recent real‐time quality tools include Framewise Integrated Real‐time fMRI Monitoring (FIRMM; Dosenbach et al., 2017), which focuses on real‐time motion tracking and related quality metrics, and rtQC, a recently presented open‐source collaborative framework for quality control methods in real‐time (Hellrung et al., 2017; Heunis et al., 2019). rtQC currently focuses on highlighting quality issues between the offline and real‐time variants of fMRI data as well as real‐time visualisation of quality control metrics, including a real‐time display of a grayplot (a 2D representation of voxel intensity fluctuations over time; Power, 2017).

*Quality reporting practices*: In further rtfMRI‐NF literature, studies employing data quality checks focus on pre‐ and post‐real‐time application of quality control processes. Stoeckel et al. (2014) propose the calculation and use of tSNR and the concordance correlation coefficient on pilot data to determine, respectively, whether the rtfMRI‐NF signal is detectable and reproducible between runs. They also proposed a list of seven high‐level guidelines to help optimise real‐time fMRI neurofeedback for therapeutic discovery and development: (a) the rtfMRI signal is accurate and reliable, (b) rtfMRI neurofeedback leads to learning, (c) the training protocol is optimised for rtfMRI‐based neurofeedback and learning, (d) there is an appropriate test of training success, (e) rtfMRI neurofeedback leads to behavioural change, (f) an appropriate rtfMRI neurofeedback‐based clinical trial design is in place and (g) sharing resources and using common standards. Sorger, Kamp, Weiskopf, Peters, and Goebel (2018) provided a list of five criteria used for selection of custom feedback ROIs per subject: including (a) robust and typical hemodynamic response shown in ROI, (b) high tSNR and tCNR, (c) ample evidence for the ROI's involvement in the selected activation strategy, (d) insensitivity to susceptibility artefacts and (e) the ROI should consist of 10–15 neighbouring voxels spanning three fMRI slices. As post‐real‐time quality control, Koush et al. (2012) report the use of four quality metrics to evaluate their real‐time denoising algorithms (tSNR, event‐related tSNR, tCNR and statistical *t*‐values), while Zilverstand et al. (2017) used mean displacement and tSNR to investigate offline data quality differences between control and test groups. Thibault et al. (2018) suggested a list of best practices for rtfMRI‐NF studies spanning the whole process from study design to outcome measurement, including suggestions for: (a) study pre‐registration, (b) sample size justification, (c) inclusion of control neurofeedback measures, (d) inclusion of control groups, (e) collection and reporting of the BOLD neurofeedback signal, (f) collection and reporting of behavioural data and (g) outcome measure definitions and reporting.

In this work, we propose both wider adoption of such best practices in rtfMRI‐NF, as well as more granular specification of data quality measurement and reporting concerning the processing steps that could influence the quality of the signal being regulated.

### 3.2 Data quality challenges in rtfMRI‐NF

Real‐time fMRI is plagued by the same noise fluctuations and artefacts present in conventional task‐based and resting state fMRI with the main difference being the required real‐time removal of these confounds per volume, versus offline otherwise. This has to be achieved with an altered technical setup compared to the conventional approach. This time‐constrained and technically novel scenario brings about a range of challenges, discussed below.

#### 3.2.1 Inseparability of data measures and subject regulation effects

A major challenge in assessing neurofeedback signal quality is the inherent mediation of the real‐time signal by the process of neurofeedback training. This mediation effect, and in fact neurofeedback learnability itself, is highly variable within and between subjects and unlikely to be estimated or predicted accurately. This is known from neurofeedback based on electroencephalography (EEG), is referred to as the inefficacy problem (Alkoby, Abu‐Rmileh, Shriki, & Todder, 2018), and appears to generalise across neuroimaging modalities. An estimated 15–30% of subjects are unable to learn control over brain computer interfaces (BCIs; Vidaurre & Blankertz, 2010), while in a review of psychological factors influencing neurofeedback learning outcomes, Kadosh and Staunton (2019) found attention, amongst other factors, to be crucial for neurofeedback learning success. The inability to reliably separate the rtfMRI signal into BOLD regulation effects versus noise (or noise‐absent signal) makes standard quantitative measures like tSNR ill‐suited to granularly assess the quality of the neurofeedback signal. Alternative measures or procedures become necessary, an example being the framework for offline evaluation and optimization of real‐time neurofeedback algorithms recently put forward by Ramot and Gonzalez‐Castillo (2019).

#### 3.2.2 Decreased statistical power

In offline fMRI denoising, data for the whole session is available and there is effectively no time limit on the processing, which respectively allows improved statistical power for noise detection and the execution of complex algorithms to model and remove noise fluctuations. Conversely, in rtfMRI‐NF the statistical power is decreased, specifically in a moving window approach or during the start of a cumulative approach due to the small amount of data samples available. Additionally, the available calculation time in real‐time is limited to the span of a single TR (in the standard case of continuous feedback), albeit mostly with fewer data to process. This means that rtfMRI algorithms can less likely detect true BOLD effects (or noise effects) as they occur, resulting in diminishing quality control of the rtfMRI‐NF signal.

#### 3.2.3 Lack of readily available peripheral measurements

Most scanner setups require custom modifications to hardware and/or software in order for extra physiological information to be transferred in real‐time. For example, to our knowledge few reports exist of physiological data (respiration and heart rate) being transferred and incorporated into a rtfMRI‐NF software pipeline to remove physiological noise in real‐time (e.g. Bodurka et al., 2009; Hamilton et al., 2016; Misaki et al., 2015). Addressing this challenge (technologically and algorithmically) could potentially be of substantial benefit to the quality of the neurofeedback signal, as it would diminish the possibility of subjects being trained on physiological nuisance signals (e.g. respiration effects) and would thus increase the contingency of the signal on actual brain activity.

#### 3.2.4 Difficulty of real‐time visual quality control

The neurofeedback signal is calculated and fed back to the subject immediately after the relevant pre‐processing and analysis has been completed within a single TR, that is, there is no time for an expert to inspect the volume, to assess its quality, and to perform conditional denoising steps, as opposed to offline fMRI quality control. However, this challenge provides an opportunity for rtfMRI‐NF to improve computational/methods reproducibility, because a potential solution would be to have automated data quality inspection and control per volume. An example would be calculating framewise displacement (FD; Jenkinson, Bannister, Brady, & Smith, 2002; Power et al., 2012) per volume using real‐time volume realignment (or head motion) parameters and automatically classifying the volume as a motion outlier or not based on some predetermined FD threshold. These outliers, in turn, could be added to a real‐time motion outlier regressor in a cumulative or incremental GLM, to achieve real‐time scrubbing, the results of which could be inspected and compared to offline counterparts after the rtfMRI experiment. Such functionality is currently available in rtQC (Heunis, Hellrung, et al., 2019).

#### 3.2.5 Differences in quality between real‐time and offline fMRI

Differences can occur in fMRI data that are reconstructed and transferred in real‐time compared to offline exported data, including changes to spatial, image orientation, image intensity and temporal information. Whereas per‐volume reconstruction and export timing (and related latency and jitter) are not critical for conventional fMRI analysis, they can cause substantial delays in real‐time processing and feedback presentation. However, specific details such as the tools and software versions used for data export and the real‐time latencies are rarely reported, which complicates reproducibility of methods. Additionally, differences in voxel intensity scaling, image orientation and image header information have been reported (Hellrung et al., 2017). Such issues, if known about at all, are hardly reported by rtfMRI‐NF studies, even though it could lead to potential differences of interpretation when analysing online versus offline data. Most rtfMRI‐NF studies process data offline in order to show the effects of neurofeedback training over time, often looking at the *t*‐statistic and clustering of significantly activated voxels in a region of interest. If this analysis is carried out on different datasets because of online‐offline quality control issues, conclusions could vary.

Several methods, applied during acquisition and data processing phases as well as offline, have been reported to decrease the detrimental effects of known fMRI noise and artefacts on the quality and SNR of the real‐time BOLD signal. The next section investigates a set of 128 rtfMRI‐NF studies to determine the prevalence of a variety of pre‐processing steps in real‐time fMRI pipelines, while the section thereafter focuses on the methods that address, at least in part, some of the above challenges.

## 4 DENOISING IN REAL‐TIME FMRI NEUROFEEDBACK STUDIES

A recent critical systematic review by Thibault et al. (2018) assessed 99 rtfMRI‐NF studies in order to evaluate the effectiveness of reported experimental protocols to train subjects to self‐regulate their BOLD signal and to induce behavioural improvements. The list featured a prominent set of the most recent rtfMRI‐NF studies spanning a variety of patient groups and feedback signal types, and also included all 12 studies used by Emmert et al. (2016) in one of the only rtfMRI‐NF meta‐analyses conducted to investigate the mechanism of brain regulation resulting from neurofeedback. Apart from its main findings, the review by Thibault et al. (2018) showed that 62 out of 99 studies did not report any account being taken of respiratory confounds, that 19 studies subtracted activity in a background region to account for so‐called global effects, and that nine studies regressed out respiration‐related noise signals in real‐time. Respiration is known to be a source of global BOLD fluctuations and its removal is seen as a recommendable pre‐processing step in conventional resting state fMRI processing (Bright & Murphy, 2013).

**FIGURE 6:**
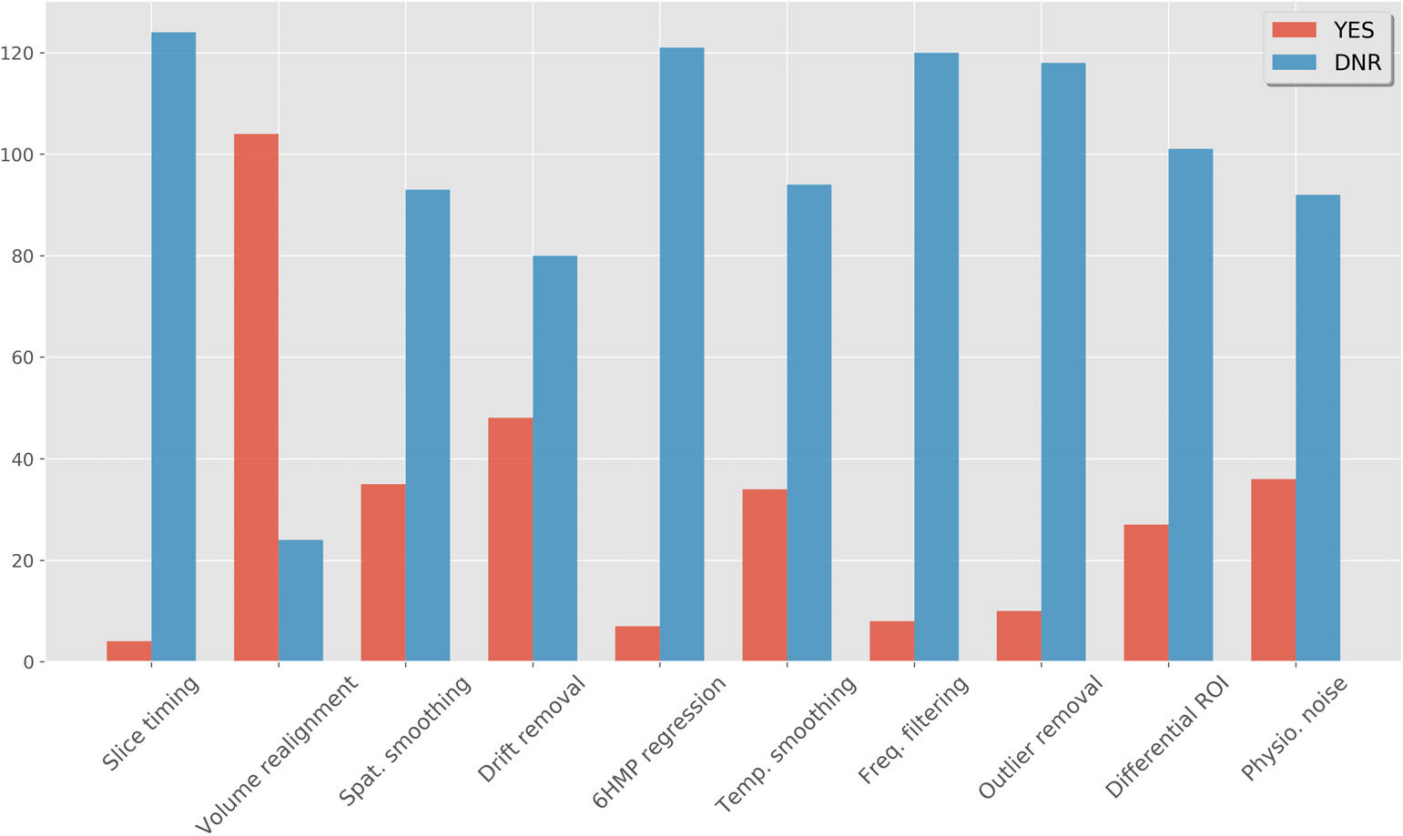
A list of real‐time pre‐processing and denoising steps used in 128 recent rtfMRI neurofeedback studies. (All bars are indicated as YES/red and DNR/blue while the breakdown for the bar ‘Differential ROI’ is 27 YES, 100 ‘DNR’ and 1 ‘No’, Marins et al., 2015). DNR, did not report

**FIGURE 7:**
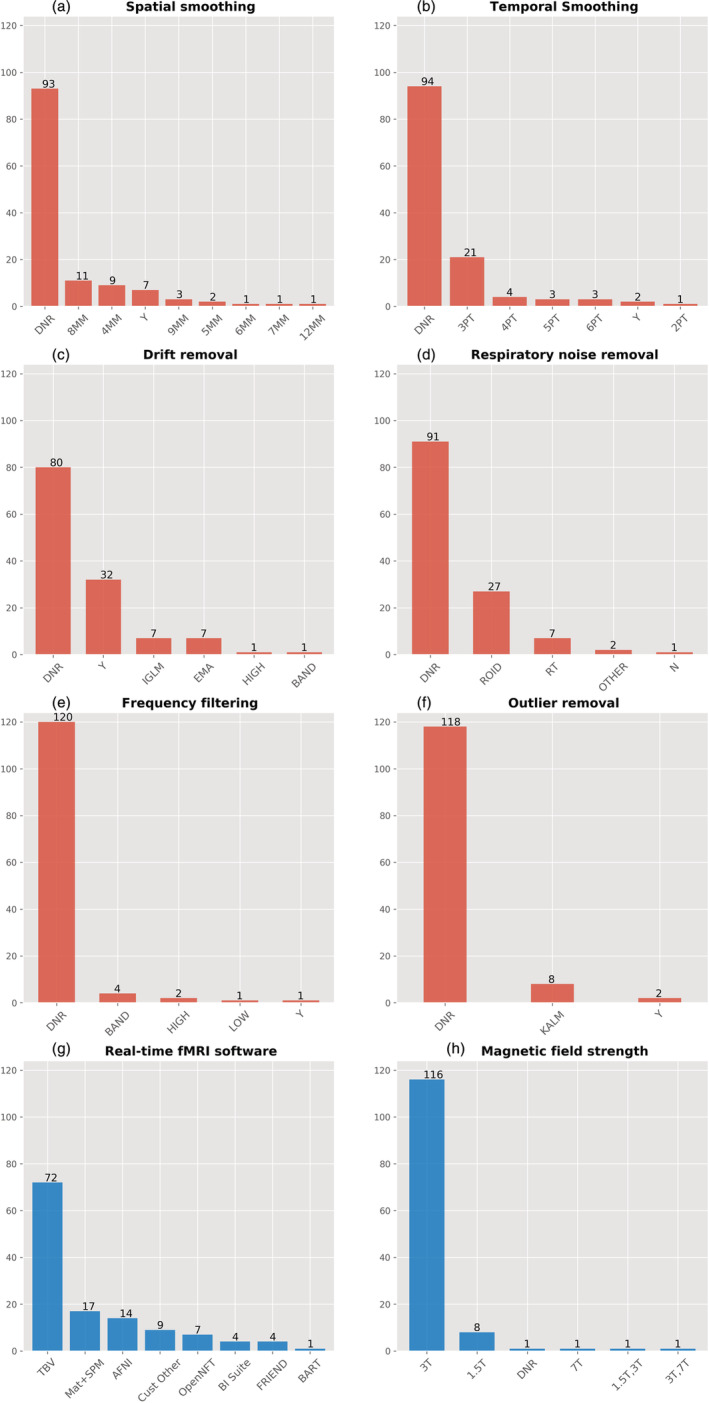
Bar graphs showing a breakdown of methods used for specific pre‐processing and/or denoising steps in the 128 studies compiled in this work (red). The last two bar graphs (blue) indicate a breakdown of other features of the studies. (a) Spatial smoothing (4,5,6,7,8,9,12MM = FWHM size of Gaussian smoothing kernel). (b) Temporal smoothing through time point averaging (2,3,4,5,6PT = number of time points used). (c) Drift removal (EMA = exponential moving average filter; IGLM = incremental general linear model; BAND, HIGH = filter types). (d) Respiratory noise removal (ROID = differential region of interest; RT = real‐time; OTHER = other methods including averaged compartment signal regression, e.g. white matter and/or ventricle signals). (e) Frequency filtering in addition to drift removal (BAND, HIGH, LOW = filter types). (f) Outlier removal (KALM = modified Kalman filter implemented in OpenNFT). (g) rtfMRI‐NF software toolboxes (TBV = Turbo‐BrainVoyager). (h) Magnet field strengths. DNR, did not report; N, no; Y, yes, but no further detail reported

To facilitate further meta‐analyses and systematic reviews, studies should not only ensure a high level of data quality (in terms of the real‐time BOLD and neurofeedback signals) but also have to consistently and comprehensively report their use of acquisition and processing methods. A further search of rtfMRI‐NF literature (including methods reviews) showed that rtfMRI‐NF processing methodology has been covered in some detail (e.g. Bagarinao, Nakai, & Tanaka, 2006; Caria, Sitaram, & Birbaumer, 2012; Weiskopf et al., 2004), but that real‐time fMRI denoising methods have not received similar attention on a more granular level. To quantify the extent to which rtfMRI‐NF studies report correcting for commonly known fMRI noise sources and artefacts, we investigated whether 128 recent studies (available at http://bit.ly/rtfmri-nf-zotero-library) reported the use of a standard list of real‐time pre‐processing steps. We conducted a Web of Science search across *All Databases* on April 9, 2019 using the same search terms and selection criteria as provided by Thibault et al. (2018), and found another 29 studies in addition to the original 99. The list of pre‐processing steps was selected based on established practices in conventional task‐based and resting state fMRI (Poldrack, Mumford, & Nichols, 2011), as well as through identifying steps specific to rtfMRI‐NF during the process of reviewing the 128 studies and further literature. The full text of each article, including supplementary material, were searched and coded for the following key terms: averag*, *band*, *cutoff*, *difference*, *differential*, *drift*, *filter*, *frequency*, *heart*, *high*, *linear*, *low*, *motion*, *movement*, *nuisance*, *outlier*, *parameter*, *pass*, physiol*, respir*, *retroicor*, *scale*, *scrub*, *slice*, *smooth*, *spike*, *trend*. All study DOIs and coded pre‐processing steps are available as part of the accompanying Supplementary Material (JSON file, Tab Delimited Text files and Notes). Data and code necessary to reproduce Figures 6 and 7 are available on Github (https://github.com/jsheunis/quality-and-denoising-in-rtfmri-nf), which also links to an interactive environment allowing exploration and visualisation of the study data.

Figure 6 shows the list of pre‐processing and denoising steps and the amount of studies that report employing these methods. Importantly, we classified studies as *Did Not Report* (DNR) if no mention of the particular method was made in the article or supplementary material, and if we could not confidently infer its use from studying the particular article's content. A possible exception to this rule is volume realignment, which could reasonably be expected to be used in almost all recent rtfMRI‐NF studies. Figure 6 shows that 24 out of 128 studies did not report applying volume realignment, and through investigating toolbox use (for a full distribution see Figure 7e) it was found that the majority of these used Turbo‐BrainVoyager (TBV; Brain Innovation, Maastricht, The Netherlands), which does allow including this as a standard real‐time pre‐processing step. Similar discrepancies could be expected in the classification of studies as DNR for any of the other denoising steps, although such discrepancies are expected to decline with the reported use of more non‐standard or novel techniques (e.g. real‐time physiological noise regression). To account further for possible discrepancies in the reporting of assumed default processing steps, we recoded the dataset such that studies that used mature and widely used software packages reflected default options where particular steps were not reported. The motivation for this step, the resulting figures, and accompanying limitations can be viewed in the Supplementary Notes. These findings highlight the importance of correctly reporting rtfMRI‐NF denoising methods, so as to promote methods reproducibility.

Further results in Figure 6 show that volume realignment is the only step reported to be used by over half of the studies, while less than half report implementing linear drift removal and less than a third report the use of spatial smoothing, temporal smoothing and outlier removal. A special case of real‐time denoising is correction for physiological noise, where multiple approaches have been used (Figure 7e). An often‐used method is differential feedback (Weiskopf, Mathiak, et al., 2004; see Section 5.2.8 for a full description), based on the assumption that global effects caused by respiration will be cancelled out when subtracting the averaged signal in a task‐unrelated ROI from the main ROI used for neurofeedback. This also assumes that global respiration effects in both areas are identical. Still, two thirds of the studies do not report any correction for physiological noise (either in real‐time or offline), while six studies use modelled physiological noise regression or data driven methods to remove noise fluctuations possibly caused by subject physiology (Thibault et al., 2018, reported nine due to a mischaracterization of offline physiological noise regression as real‐time regression in some cases). Additionally, it was also found that while several studies reported the use of optimised acquisition sequences to reduce susceptibility‐induced image distortion (e.g. the spiral‐in/out sequence by Glover & Law, 2001), only one study from the 128 reported incorporating post‐acquisition distortion correction into their real‐time algorithm (Marxen et al., 2016).

Finally, Thibault et al. noted a lack of both pre‐registration of rtfMRI‐NF study designs and registered report type publications, as well as a lack of adoption of general open science principles. Although open source software solutions like AFNI's real‐time plugin (Cox et al., 1995), BART (Hellrung et al., 2015), FRIEND (Basilio et al., 2015) and OpenNFT (Koush et al., 2017a) counter some of these concerns, we find additionally that minimal evidence exists for open data and methods sharing. Specifically, apart from a large dataset on default‐mode‐network neurofeedback shared publicly by McDonald et al. (2017), a useful single‐subject dataset for testing OpenNFT functionality and general methods development (Koush et al., 2017c), and further useful supplementary data shared in some cases (e.g. Zilverstand et al., 2017), we found no other publicly available rtfMRI‐NF datasets related to the investigated studies.

## 5 METHODS TO IMPROVE SIGNAL QUALITY AND DENOISING IN REAL‐TIME FMRI NEUROFEEDBACK

This section addresses the third research question of this review: *which methods for denoising data and improving data quality and SNR are available to the researcher studying rtfMRI‐NF?* We consider acquisition methods and processing methods, focusing on the latter, and investigate current rtfMRI‐NF algorithms and their capabilities with regards to the reported noise mitigation or denoising methods. Some offline methods for neurofeedback signal quality checking, although not strictly real‐time, are also considered.

### 5.1 Acquisition methods

As with conventional fMRI, it is recommended that researchers take the necessary precautions to mitigate the introduction of any unwanted noise sources into the data. This includes the possibility of using physical interventions—e.g. individualised head restraints (https://caseforge.co/), bite bars, foam pads or end‐tidal forcing systems—to counter head motion or respiratory rate variation artefacts, respectively, but also extends to tweaking pulse sequence parameters or implementing alternative sequences to increase BOLD sensitivity. Several pulse sequences, hardware changes and other acquisition steps are highlighted below.

#### 5.1.1 EPI, acceleration and high field imaging

The gradient echo EPI sequence still remains the most widely used technique for real‐time fMRI, as it allows fast acquisition of volumes covering the whole brain. The main disadvantages of the EPI sequence are that it is sensitive to susceptibility effects and machine instabilities, although all major vendors offer techniques that compensate (partly) for these scanner effects.

The EPI sequence also allows for the acquisition of multiple echoes. The basic advantage of multi‐echo over standard single‐echo EPI is that it allows more data to fit an assumed mono‐exponential decay curve, which can yield voxel‐wise estimations of *S*_0_ (initial signal intensity, that is, magnetization) and *T*_2_* (transverse relaxation time, Posse et al., 1998). Increased BOLD sensitivity results when these spatially varying *T*_2_* values are combined so as to leverage optimal BOLD contrast at each voxel, as opposed to assuming a single *T*_2_* for all grey matter voxels as per single‐echo EPI processing. Posse et al. (1999) incorporated this advantage into their ‘TurboPEPSI’ imaging technique for spectroscopic imaging, and later adapted it into the FIRE software toolbox for use in real‐time fMRI (Posse et al., 2000), using linear echo summation. Further improvements led to the development of a method, using TurboPEPSI, for quantitative *T*_2_* mapping as well as compensation of susceptibility related signal losses in multiple brain regions at different echo times (Posse, Shen, Kiselev, & Kemna, 2003). Later, Weiskopf, Klose, Birbaumer, and Mathiak (2005) implemented a real‐time multi‐echo EPI acquisition sequence that corrected for dynamic distortions along the phase‐encoding direction without the need for additional reference scans (as per standard static B0 field correction techniques).

The EPI sequence can also be combined with various accelerations techniques. The introduction of parallel imaging techniques, for example, SENSE and GRAPPA, has contributed significantly to improved spatial resolution in fMRI, and has become standard in recent imaging applications. Also, advancements in acquisition speed using multi‐band or 3D EPI techniques can improve temporal resolution as well as real‐time SNR characteristics, for example, as demonstrated by the multi‐slab echo volumar imaging techniques of Posse et al. (2012) implemented in real‐time. It should be noted, however, that the possible improvement in temporal resolution resulting from multi‐band sequences must be balanced with the possible increase in image reconstruction time required if implemented on default scanner hardware.

Lastly, imaging at higher field strengths can improve SNR and BOLD sensitivity (Triantafyllou et al., 2005), both of which is beneficial to real‐time fMRI neurofeedback. High field imaging at 7 T could be particularly useful to overcome the lower SNR provided by 1.5 and 3 T imaging in sub‐cortical regions, as demonstrated by Sladky et al. (2013) for the amygdala, and by Hahn et al. (2013) for the insula. However, an important consideration for neurofeedback at 7 T is that physiological noise increases with field strength and may dominate the BOLD signal of interest (Krüger & Glover, 2001), necessitating an appropriate denoising procedure. If accurately accounted for, however, the increased BOLD sensitivity at 7 T could improve the quality of the neurofeedback signal, which would allow closer examination of the hypothesised coupling between learning effects and the neurofeedback signal. rtfMRI‐NF at 7 T has been implemented (e.g. Andersson et al., 2011; Hollmann et al., 2008) and compared to rtfMRI‐NF at 3 T (Gröne et al., 2015). The latter study found slightly greater increases in post‐neurofeedback ROI activation in the 3 T subject group compared to the 7 T group. The difference was ascribed to a decrease in tSNR in the 7 T group compared to the 3 T group, due to several contributing factors including shimming conditions, B1‐field inhomogeneities, phase‐encoding polarity and physiological noise.

#### 5.1.2 Alternative sequences and shimming

Alternative sequences or shimming practices have also been used to minimise the real‐time image distortion or dropout artefacts related to local susceptibility gradients or other causes. A sequence developed by Glover and Law (2001) follows a spiral in/out readout trajectory of k‐space that reduces signal dropout and increases BOLD contrast. Spiral‐in has the advantage that it allows for higher temporal resolution, while spiral‐out allows for short echo times which could also be an advantage in multi‐echo denoising applications (e.g. when regressing the short echo signal out of the acquired data to remove proximal *S*_0_ effects; Bright & Murphy, 2013). Spiral in/out acquisition has been implemented by a number of rtfMRI‐NF studies (Greer, Trujillo, Glover, & Knutson, 2014; Hamilton et al., 2016; Hamilton, Glover, Hsu, Johnson, & Gotlib, 2010). Real‐time shimming to account for geometric distortion has also been implemented. Here, Ward, Riederer, and Jack (2002) implemented a sequence to detect and correct for linear shim changes in real‐time, while van Gelderen, de Zwart, Starewicz, Hinks, and Duyn (2007) used a reference B0 scan and chest motion data to apply respiration‐compensating B0 shims in real‐time.

#### 5.1.3 Prospective motion correction and motion feedback

In addition to various MRI acquisition methods, prospective motion correction is another step that could increase SNR of the rtfMRI BOLD signal during the acquisition phase. Image‐based motion detection (Thesen et al., 2000), field cameras (Dietrich et al., 2016) or external optical tracking methods (Zaitsev et al., 2006) have been used to estimate rigid body transformations and subsequently update pulse sequence parameters in real‐time, such that the imaging volume essentially ‘follows’ the subject's movement (Maclaren, Herbst, Speck, & Zaitsev, 2012). Another method to curtail subject head motion is to feed back the head motion parameters (HMPs), derived from real‐time head motion correction algorithms, to the subject. This in itself is a form of biofeedback training, and has been shown to reduce subject motion during scanning. In the case of Yang et al. (2005), HMPs resulting from real‐time motion correction of functional image volumes were presented to subjects in the form of a composite ‘head motion index’, similar to framewise displacement. Greene et al. (2018) used FD as the feedback measure in their implementation using FIRMM software. Importantly, the implications of feeding back several measures to the subject and displaying them together with task instructions have to be properly understood and weighed before deciding on its use.

#### 5.1.4 Adaptive paradigms

Adaptive paradigms provide interventions at a variety of stages in the acquisition and processing pipeline and allow selective data acquisition or presentation based on subject‐specific measures and a predefined set of criteria. For example, Wilms et al. (2010) developed a system where real‐time eye‐tracking data could be used to generate gaze‐contingent stimuli during fMRI experiments, while Hellrung et al. (2015) created an integrated, open‐source framework for adaptive paradigm design, which also allows the dynamically updated design (based on a gaze direction‐contingent assessment in their pilot experiment) to be transferred to the real‐time GLM for adaptive processing. Such interventions could improve data quality and SNR by earmarking the volumes during which the subject adhered to selected quality control criteria.

#### 5.1.5 Peripheral data collection

Lastly, the collection of peripheral subject data (e.g. heart rate, respiration rate, motion/eye tracking) for denoising purposes is strongly recommended. While it may not always be possible to correct for physiological noise or motion in real‐time using these measures (given technical constraints or other reasons, see below) they should at least be used offline to calculate and comment on correlations with BOLD fluctuations, task design or other subject‐related or experimental confounders.

### 5.2 Processing methods

As previously mentioned, real‐time denoising methods tend to follow the course of standard offline fMRI pre‐processing, although reports on the use of individual steps vary. Section 4 provided a list of real‐time pre‐processing or denoising steps employed by recent rtfMRI‐NF studies, with Figures 6 and 7 indicating their relative usage. These methods are presented below together with other techniques identified through further literature search.

To aid the reader's understanding and guide future use of these methods, Table 1 summarises the most often reported real‐time processing methods. In addition, Table 1 provides context for analogous methods in conventional fMRI analysis, how the real‐time methods differ from their offline counterparts, and recommendations for deciding on implementation. Table 1 focuses *processing methods* since there would mostly be no differences between *acquisition methods* implemented to improve data quality for real‐time versus conventional fMRI. It should be noted, however, that some artefacts like electromagnetic spikes or shimming errors could be of greater importance for real‐time analysis compared to conventional fMRI analysis, since they could possibly be compensated for offline. This possibility does not exist for neurofeedback applications, hence extra care should be taken at the acquisition stage to try and avoid such artefacts. Table 1 also lists possible approaches to this challenge using real‐time processing methods.

**TABLE 1:** A summary of real‐time fMRI neurofeedback processing methods

Conventional method	Real‐time method	Differences/notes	Real‐time recommendation
***0. Example processing step***			
*Standard method(s) used in conventional offline fMRI processing*	*Methods most often used or reported in real‐time fMRI and neurofeedback signal processing*	*Main distinctions between offline and online/real‐time methods, with additional notes*	*Recommendations on the use of the reported real‐time methods, whether to implement them or not and additional relevant information*
***1. Slice timing correction***			
Various interpolation methods	Various interpolation methods	No algorithmic differences, as both are done on a per‐volume basis	Generally recommended for TR ≥2 s
***2. 3D volume realignment***			
6 degree of freedom rigid body transformation of whole brain data	6 degree of freedom rigid body transformation of whole brain data	No algorithmic differences, as both are done on a per‐volume basis Template EPI for real‐time = previously collected EPI volume Template EPI for offline = first volume of time‐series or mean EPI	Always recommended
***3. Spatial smoothing***			
3D Gaussian smoothing kernel with a specified FWHM, applied to whole or masked brain data	(3.1) 3D Gaussian smoothing kernel with a specified FWHM, applied to whole or masked brain data	No algorithmic differences, as both are done on a per‐volume basis	Typically recommended to increase SNR for all except MVPA‐based neurofeedback methods or when using small ROIs (e.g. amygdala) Recommended kernel size depends on acquisition parameters amongst multiple other factors
	(3.2) Averaging voxel values within a pre‐specified ROI	Kernel‐based smoothing versus basic averaging	Typically recommended to allow calculation of a 1D neurofeedback signal from 3D data
***4. Drift removal and frequency filtering***			
Various algorithms for (mostly) high‐pass filtering, for example, a cosine basis set as GLM regressors (SPM) or a Gaussian‐weighted running line smoother (FSL). Typically applied to whole or masked brain data. Low‐pass, band‐pass or other types of filtering typically applied as part of GLM.	(4.1) Incremental GLM (iGLM) with filtering regressors, for example, a cosine basis set and/or a linear trend regressor	GLM applied to full offline data versus real‐time iGLM Whole brain offline drift removal (filtering) versus drift removal from 1D neurofeedback signal in real‐time	Drift removal is always recommended Piloting suggested to determine the method best suited for the data Kopel et al. (2019) recommend a sliding window iGLM algorithm with standard cosine basis set PSC calculation is always with reference to a baseline. Thus, inherent drift removal through baseline subtraction is recommended; if not a global mean, then least ROI‐based; if not cumulative, then at least based on the preceding baseline (non‐regulation) block.
	(4.2) Exponential moving average (EMA) filter	No algorithmic differences if applied as digital filter that takes only history into account Whole brain offline drift removal (filtering) versus filtering of 1D neurofeedback signal in real‐time	
	(4.3) Inherent baseline drift removal through subtraction of cumulative global mean from ROI signal during PSC calculation	Mostly limited to real‐time application because of PSC calculation for neurofeedback. *See related: global signal regression in 6 below*	
***5. Temporal filtering or averaging***			
Typically, this is an implicit result of filtering as described above	(5.1) Moving window time‐point averaging of 1D neurofeedback signal	Standard offline filtering versus online 1D signal time‐point averaging (comparable to EMA filter)	Piloting suggested to determine if time‐point averaging is suitable Three‐point window size is most common in studies that reported implementing this method
An AR(1) filter is typically, and explicitly, applied to address autocorrelation in fMRI time‐series data	(5.2) AR(1) filtering in real‐time has been reported but seldom implemented	No algorithmic differences if applied as digital filter per time‐point that takes only history into account	Piloting suggested to determine if AR(1) filtering is useful in addition to and/or influenced by other standard temporal smoothing and filtering steps
***6. Nuisance regression (excluding physiological noise removal)***			
Various 1D data traces are often included as nuisance regressors in offline GLM, including: Head movement parameters (HMPs) Volterra expansion of HMPs Tissue compartment signal averages (CSF, WM, GM) Global signal Typically applied to whole or masked brain data	Incremental GLM (iGLM) with minimal filtering regressors, for example: Head movement parameters (HMPs) Tissue compartment signal averages (CSF, WM, GM, global)	GLM applied to full offline data versus real‐time iGLM Whole brain offline nuisance regression, versus nuisance regression from 1D neurofeedback signal in real‐time	Piloting suggested to determine which nuisance regressors are best suited for the data Over specification of design matrix (i.e. too many regressors) is not recommended, as iGLM parameter estimates will be noisy and will take considerable time to stabilise (see Misaki et al., 2015) Global signal regression is controversial in offline and real‐time fMRI analysis and should be piloted and well justified
***7. Outlier or spike removal***			
‘Scrubbing’ low quality EPI volumes (removing, replacing, averaging) based on a variety of quality metrics, for example: Framewise displacement DVARS *SD* Z‐score Other Could be incorporated as an additional scan‐nulling regressor in offline GLM	(7.1) Possibility to do real‐time scan‐nulling as part of iGLM, for example, through real‐time outlier detection based on a predefined framewise displacement threshold	GLM applied to full offline data versus real‐time iGLM Whole brain offline nuisance regression, versus nuisance regression from 1D neurofeedback signal in real‐time Detection thresholds set based on statistical properties of full dataset or group data, versus requirement for predefined threshold for real‐time detection	Piloting suggested to determine if outlier removal is useful, and whether other existing filtering methods (e.g. iGLM regressors, EMA, temporal smoothing) could suffice Careful thought should be given to predefined detection threshold if real‐time outlier detection and scan‐nulling is considered Kalman filter parameters should be piloted, and defaults should not be accepted as best for the data
	(7.2) Kalman filter that detects and rejects outliers based on irregular statistical properties (Koush et al., 2012)	Standard high/low/band‐pass filtering is typically used offline on whole brain data Adaptive Kalman filter introduced for real‐time and implemented on 1D neurofeedback signal	
***8. Physiological noise removal***			
Physiological noise is typically modelled using concurrent recordings of respiration and heart rate, for example, RETROICOR, RVT, HRV. These are then used as nuisance regressors in the offline GLM applied to whole brain data	(8.1) Incremental GLM (iGLM) with additional filtering regressors, for example: RETROICOR set Tissue compartment signal averages (CSF, WM, GM, global)	GLM applied to full offline data versus real‐time iGLM Whole brain offline nuisance regression, versus nuisance regression from 1D neurofeedback signal in real‐time	Piloting suggested to determine which nuisance regressors are best suited for the data Over specification of design matrix (i.e. too many regressors) is not recommended, as iGLM parameter estimates will be noisy and will take considerable time to stabilise (see Misaki et al., 2015) Given the additional technical challenge of processing physiology traces in real‐time, RETROICOR nuisance regression is not recommended unless pilot data or new evidence suggest otherwise
	(8.2) Differential ROI to (potentially) correct for global effects caused by respiration	An analogous step to real‐time differential ROI does not exist for standard offline analysis Differential ROI calculations are based on 1D ROI‐averaged signals	Piloting suggested to determine whether this is suited for the data Care should be taken to ensure that task‐relevant information is not subtracted from the More evidence is to be gathered before this could be considered a recommended real‐time processing step, or not
	(8.3) High frequency filtering or adaptive Kalman filtering	Standard high/low/band‐pass filtering is typically used offline on whole brain data Adaptive Kalman filter introduced for real‐time and implemented on 1D neurofeedback signal	Kalman filter parameters should be piloted, and defaults should not be accepted as best for the data
***9. Signal scaling***			
Global, proportional, and/or grand mean scaling steps are often applied to whole brain time‐series data (e.g. prior to first level analysis in SPM and FSL), which typically allows the validity of analyses between runs and subjects	Signal scaling is most often done on the ROI‐averaged 1D neurofeedback signal, taking historical time‐series values into account. Scaling methods include: Temporal smoothing, as described above in 5 Using a dynamically updated range based on prior time‐series data	Whole brain intensity scaling to allow comparisons across runs/subjects versus scaling the 1D neurofeedback signal to prevent abrupt changes to the display seen by the subject	Real‐time signal scaling for visual quality of the neurofeedback signal is recommended The specific scaling method should be determined through piloting
***10. Model free denoising methods***			
Principal and/or independent component analysis is often applied to whole brain time‐series data in order to extract statistically independent spatial components. These components can be classified as noise sources and subsequently regressed from the whole brain time‐series data. Examples include: MELODIC ICA ICA‐AROMA aCompCorr	Model free methods are generally not reported in real‐time fMRI analysis, although examples exist (Esposito et al., 2003)	ICA is generally time‐consuming and requires (without some form of regularisation) full datasets in order to generate useful noise components. This is a technical challenge for real‐time implementation	ICA‐based methods for real‐time denoising are generally not recommended unless new algorithms are developed with accompanying evidence that suggests otherwise
***11. Offline quality checking***			
This offline step serves to report features of the data that can be (often visually) inspected and compared to thresholds in order to assess the overall quality of spatial and time‐series aspects of whole brain datasets. Useful tools and metrics include: MRIQC QAP Framewise displacement DVARS Timeseries plots	For real‐time fMRI, offline quality checking steps are not standardised and hardly reported. A minority of studies investigate possible correlations between physiology or motion traces and the neurofeedback regulation paradigm	There would essentially be no difference between standard tools and metrics for offline quality control of full datasets and post hoc real‐time datasets, as long as it is done on data as exported from the scanner in a standard way, since real‐time exported data might contain differences	Offline data quality checking and reporting is always recommended, especially with regards to sources of variance that could not sufficiently be corrected for in real‐time but could still skew the neurofeedback learning outcomes. Examples include: Reporting correlations between head movement parameters and the neurofeedback regulation paradigm Reporting correlations between physiology traces (and derived RETROICOR regressors) and the neurofeedback regulation paradigm Implementing physiological noise correction in post hoc analyses

Abbreviations: AR, autoregressive; CSF, cerebrospinal fluid; DVARS, differential variance root mean squared; EMA, exponential moving average filter; EPI, echo‐planar imaging; FWHM, full width half max size of Gaussian smoothing kernel; FSL, software library; GLM, general linear model; GM, grey matter; HMP, head movement parameter; HRV, heart rate variability; ICA, independent component analysis; iGLM, incremental general linear model; MVPA, multivariate pattern analysis; PCA, principal component analysis; RETROICOR, retrospective image‐based correction; ROI, region of interest; RT, real‐time; RVT, respiratory volume per time; SNR, signal‐to‐noise ratio; SPM, software library; TR, repetition time; WM, white matter; Y, yes.

#### 5.2.1 Distortion correction

Geometric distortion effects, if addressed, are mostly accounted for using specialised acquisition methods as presented in the previous section, although correction through real‐time processing is possible. An example is the point spread function (PSF) mapping approach developed by Zaitsev, Hennig, and Speck (2004) that is used in combination with parallel imaging techniques to allow fast and fully automated distortion correction of EPI. Note that this was implemented on scanner infrastructure and not as part of an external real‐time fMRI software toolbox, and that it requires a reference PSF map scan. This method was used in a rtfMRI‐NF study at 3 T with multi‐echo EPI by Marxen et al. (2016). Another example is the dynamic, multi‐echo distortion correction sequence implemented by Weiskopf et al. (2005), also implemented on scanner infrastructure.

#### 5.2.2 Slice timing correction

Slice timing correction interpolates the data of different 2D slices acquired at slightly different time points along the hemodynamic response, such that the resulting 3D image represents brain activity sampled at the same time point (Sladky et al., 2011). It has been suggested that event‐related analysis in fMRI is relatively robust to possible slice timing problems in sequences with a TR ≤2 s (Poldrack et al., 2011). With dynamic causal modelling (DCM), whose initial formulations assumed a single time point sampling of all 2D slices in an fMRI volume, Kiebel, Klöppel, Weiskopf, and Friston (2007) showed with simulations that exclusion of slice timing correction leads to larger deviations from the true connectivity parameters. They showed further that this problem is easily overcome by including information about temporal sampling in the dynamic causal model (explicitly as an extra model level). While Koush et al. (2017) do not include slice‐timing correction in their pipeline, they specifically mention selecting a short repetition time (1,100 ms) to limit the effects of slice‐timing differences in their implementation of DCM‐based neurofeedback.

Although some rtfMRI‐NF toolboxes allow real‐time slice timing correction through plugin or additionally developed functionality (e.g. OpenNFT, Turbo‐BrainVoyager), few rtfMRI‐NF studies report its use (Harmelech, Friedman, & Malach, 2015; Harmelech, Preminger, Wertman, & Malach, 2013), which might be explained by reporting discrepancies or by the generally short TR used in typical neurofeedback studies. To our knowledge, no studies have been conducted to determine its usefulness in rtfMRI‐NF.

#### 5.2.3 3D volume realignment

As one of the major noise sources in fMRI, head motion received much attention during initial algorithm development in real‐time fMRI. Cox and Jesmanowicz (1999) developed a fast method for 3D image registration in real‐time that was incorporated into AFNI's real‐time fMRI module (Cox et al., 1995), while Mathiak and Posse (2001) developed the EMOTIONAL FIRE algorithm to perform 3D rigid body realignment as part of the FIRE rtfMRI package (Gembris et al., 2000). Most other rtfMRI‐NF toolboxes or custom software implementations allow some form of 3D volume realignment, for example, OpenNFT (Koush et al., 2017a) which uses a faster version of SPM12's *spm_realign* routine (SPM, www.fil.ion.ucl.ac.uk/spm), or FRIEND (Basilio et al., 2015) that incorporates FSL's MCFLIRT algorithm (FSL, https://fsl.fmrib.ox.ac.uk/fsl).

Regression of the six HMP time courses (and their framewise derivatives and/or squares derived by Volterra expansion) is a typical step used in conventional fMRI pre‐processing to correct for residual motion effects (Friston et al., 1996). This can be implemented in the incremental or cumulative GLMs typically used in real‐time fMRI, and some studies have reported its use (Hamilton et al., 2016; Harmelech et al., 2013, 2015; Kim, Yoo, Tegethoff, Meinlschmidt, & Lee, 2015; Yamashita, Hayasaka, Kawato, & Imamizu, 2017).

#### 5.2.4 Spatial smoothing

Spatial smoothing of fMRI volumes with a Gaussian kernel is typically recommended to increase the SNR for detection of signals with a spatial extent larger than a few voxels (Poldrack et al., 2011). Given that the neurofeedback signal is typically derived (per volume) from averaging the signal intensity over multiple voxels within an ROI, a basic form of spatial smoothing is inherently applied. It could be argued that this negates the need for an extra spatial smoothing step in the real‐time fMRI processing pipeline, but further research is necessary to determine this argument's validity. Numerous rtfMRI‐NF studies report spatially smoothing their fMRI data before calculating the neurofeedback signal, while in some cases it might be explicitly excluded, for example, neurofeedback based on MPVA of voxels within an ROI, or for small regions of interest like the amygdala imaged at high field strengths (Sladky et al., 2018).

#### 5.2.5 Linear detrending/drift removal

Correcting for signal drift is a relatively standard step in real‐time fMRI and could form part of the real‐time GLM procedure, where a linear term and/or basis set of low frequency drift terms are included as regressors, acting as a high‐pass filter. An inherent correction for baseline drift is also executed in some percentage signal change neurofeedback paradigms during feedback signal calculation, due to the cumulative global mean being subtracted from the averaged ROI BOLD signal (e.g. deCharms et al., 2005; Garrison et al., 2013). Most major rtfMRI‐NF toolboxes allow some form of low‐frequency drift correction. In a recent study, Kopel et al. (2019) compared the performance of commonly used online detrending algorithms with regards to their ability to eliminate drift components and artefacts without distorting the signal of interest. They found performance to be similar for exponential moving average (EMA), incremental general linear model (iGLM) and sliding window iGLM (iGLM^window^), although the latter option was proposed for future studies.

#### 5.2.6 Temporal filtering or averaging

Further filtering of real‐time fMRI data is possible, for example, with the exponential moving average filter employed by Koush et al. (2012) to remove both high frequency noise and low frequency drift from the BOLD signal, or by including regressors relating to a specific frequency pass‐band in the real‐time GLM. Averaging of timepoints before calculating the neurofeedback signal, using a moving window approach, is another step implemented in several rtfMRI‐NF studies (e.g. Young et al., 2014).

#### 5.2.7 Outlier or spike removal

Removal or replacement of outlier volumes or data based on some quality criteria (whether defined visually or according to data calculations) is a method employed in conventional fMRI analysis to improve SNR (Power et al., 2014). Similar steps have been taken in real‐time fMRI, for example, in the BioImage Suite and custom Matlab implementation of Garrison et al. (2013), where a volume is classified as an outlier and replaced by the previous volume if mean activation in the ROI differed by more than 10% from the previous measurement. Koush et al. (2012) implemented an adapted Kalman filter, by applying nonlinear modifications, that define outliers by their irregular statistical properties in order to achieve spike detection and high frequency filtering. This algorithm has been incorporated into the open‐source OpenNFT toolbox as part of its standard real‐time processing pipeline (Koush et al., 2017a). Additionally, the Kalman filter requires only the current datapoint and previous state information, as opposed to all previous data points (or a subset thereof), and therefore does not add much latency to the real‐time pipeline. Lastly, outlier rejection based on a standardised voxel intensity threshold has also been reported by McCaig, Dixon, Keramatian, Liu, and Christoff (2011), in which they exclude voxels with a standardised intensity of *z* < −2.0 from the real‐time ROI analysis in order to reduce noise associated with out‐of‐brain voxels and signal dropout.

#### 5.2.8 Accounting for global effects through differential feedback

Feedback on the difference signal between ROIs has been motivated as a way to cancel out global effects like global intensity changes caused by respiration‐induced artefacts (deCharms et al., 2004; Weiskopf et al., 2004; Weiskopf, Mathiak, et al., 2004). In addition to the main ROI selected for neurofeedback, a reference or background ROI is typically defined as a task‐unrelated axial slice or 3D ROI, in which the average signal is calculated and subtracted from the main ROI. Alternatively, defining the reference ROI as another task‐related region allows subjects to attempt more specific bidirectional control of brain activity due to general regulation effects being cancelled out, for example, using both the supplementary motor area and the parahippocampal place area as ROIs for PSCNef (Weiskopf, Scharnowski, et al., 2004). These points have motivated several studies to opt for differential feedback over standard (non‐differential) feedback, although a limitation would be that global effects may in fact vary substantially across the brain and that differential feedback might actually decrease SNR if activation related information is contained within the reference ROI (Marins et al., 2015). To our knowledge, no experiments have been conducted and published that investigate the relationship between differential feedback and SNR of the feedback signal, thus further research would benefit this area.

#### 5.2.9 Physiological noise correction (respiration and heart rate)

Denoising physiological confounds has been approached in a variety of ways in rtfMRI‐NF, even though most studies do not report any correction for physiological noise. In those that do, differential feedback is most often used as a potential correction method for global effects caused by respiration (although accompanied by above‐mentioned caveats). Filtering can also remove some physiology‐induced variance, with the modified Kalman filter by Koush et al. (2012, 2017a) being a special case where high‐frequency spikes resulting from changes in head position or breathing can be filtered out with no prior assumption about the specific noise model. Another option to remove physiology‐related variance is to regress the spatial‐averaged time course of compartments like white matter or the ventricles from the signal of interest, that is, a real‐time version of tissue‐based nuisance regression as conventionally used in offline analyses. Spetter et al. (2017) calculated partial correlation of areas of interest with white matter and used these results to regress out any unwanted fluctuations before the neurofeedback signal was calculated, and Yamashita et al. (2017) included averaged signals from white matter, grey matter and CSF as nuisance signals in their real‐time regression analysis.

Model‐based approaches follow the work done by Glover et al. (2000), Birn et al. (2006) and Chang, Cunningham, and Glover (2009) on retrospective image correction (RETROICOR), respiratory volume per time (RVT) and heart rate variability (HRV), respectively, where concurrent recordings of the subject's breathing and heart rate are used to create nuisance regressors used in subsequent real‐time linear modelling. With physiological signal monitoring built into AFNI's real‐time plugin (Bodurka et al., 2009), Misaki et al. (2015) implemented the first real‐time RETROICOR and RVT physiological regression as an extension, using a GPU to denoise over 100 k voxels (i.e. whole brain data) in under 300 ms per volume. Hamilton et al. (2016) reported including two physiological noise regressors in their real‐time regression analysis implemented in custom C/C++ and Matlab, with no further detail provided.

Time synchronisation of peripheral recordings and fMRI data is a legitimate challenge to model‐based correction of breathing and heart rate variability artefacts in real‐time, unless the challenge is avoided altogether by using advanced processing power and full recalculation of all available data for every iteration, as was done by Misaki et al. (2015). Some global time‐stamping solutions have been implemented to allow synchronisation of concurrent physiology and fMRI recordings (Hellrung et al., 2015; Smyser et al., 2001; Voyvodic, 2011). This typically requires a custom‐programmed software package dedicated to managing time‐synchronisation of multiple concurrent inputs and outputs, for example, the CIGAL software (Voyvodic, 1999) which could run modules in parallel for the main stimulus event, a button‐press hardware input, an analog data input for physiological recordings, the scanner trigger, eye‐tracker recordings of eye position and pupil diameter and more.

Lastly, we found no examples of studies investigating and comparing the efficacy of different real‐time physiological noise removal strategies or their effect on the neurofeedback signal in rtfMRI‐NF, although regarding offline correction, it has been suggested that motion or physiological fluctuations do not drive neurofeedback learning effects (Hellrung et al., 2018).

#### 5.2.10 Other real‐time processing methods

*Global signal regression*, although a controversial denoising step in offline fMRI processing (Murphy & Fox, 2017), can be used in real‐time to remove global fluctuations common to large areas of the brain and hypothesised to be of non‐neuronal origin. This would typically involve including the cumulative global mean signal in the real‐time GLM and regressing that out of the averaged ROI BOLD signal of interest, similar to CSF and white matter compartment regression.

*Independent component analysis* (ICA) has been a very effective tool in finding nuisance networks in resting state fMRI, which can be regressed out of the fMRI time series for effective denoising. Esposito et al. (2003) were the first to implement a real‐time ICA algorithm using a sliding‐window approach on a limited amount of axial brain slices, as a plugin to Turbo‐BrainVoyager. Although this was used to generate quasi‐stationary activation maps and accompanying time courses, this demonstration sufficed to highlight the possibility of generating the spatiotemporal characteristics of nuisance signals for real‐time denoising. This functionality, however, has not extended towards wider exploration or adoption.

*Voxel efficiency scaling* was proposed and implemented by Hinds et al. (2011) in their software toolbox Murfi as a way to avoid the undesired noise weighting resulting from standard direct averaging of all voxels within the neurofeedback region of interest. Rather, a z‐score weighted average of the ROI voxels were used for neurofeedback signal calculation, which they found to result in increased SNR of the neurofeedback signal compared to a post hoc calculation method as well as the standard direct averaging method.

Lastly, *multi‐echo EPI* processing methods in real‐time have also shown promise in increasing the SNR of the real‐time BOLD signal, specifically in areas of the brain where the local *T*_2_* is not close to the standard EPI echo time of ~30 ms selected for optimal BOLD contrast at 3 T. The multi‐echo acquisition methods reviewed earlier are typically accompanied by echo summation schemes that allow real‐time increases in BOLD sensitivity. Posse et al. (2003) implemented a fixed, linear, TE‐weighted summation of echo signals, a processing scheme later also used by Marxen et al. (2016) in their neurofeedback study of the amygdala. After multi‐echo image acquisition and real‐time distortion correction of all echo images, Weiskopf et al. (2005) used a BOLD sensitivity curve for weighted combination. Several other combination schemes are possible (e.g. Poser, Versluis, Hoogduin, & Norris, 2006), and in related work we have investigated the comparative performance of various real‐time combination schemes in terms of tSNR distributions (Heunis, Lamerichs, Song, Zinger, & Aldenkamp, 2019). Further work is necessary to determine their comparative efficacy in terms of extended quality metrics important to rtfMRI‐NF.

#### 5.2.11 Further quality control of the feedback signal

Some methods do not consist of efforts to denoise the real‐time BOLD signal of specific nuisance fluctuations, but rather to improve the quality of data acquisition or feedback presentation in real‐time. Offline methods are also used as post hoc data quality checks.

Temporally averaging and scaling the feedback signal are often used to prevent abrupt changes to the signal presented to the subject in real‐time. For example, Garrison et al. (2013) used a sliding window of five volumes for temporal smoothing of the mean ROI activation intended for neurofeedback. OpenNFT (Koush et al., 2017a) uses a dynamic range, defined by the average of the 5% highest and lowest acquired activity time points, to scale the dynamic feedback signal.

Lastly, several quality control methods have been proposed to determine whether respiration or heart rate fluctuations may have had any significant effect on the neurofeedback signal calculation that could bias the data. These should be separated from real‐time denoising algorithms which aim to remove the noise/artefact *before* the feedback signal is calculated and displayed to the subject. For example, Sorger et al. (2018) collect real‐time cardiac and respiratory traces and analyse them after the neurofeedback session to investigate possible correlations with the task design or other BOLD fluctuations. Physiological traces can also be incorporated into offline physiological denoising (e.g. RETROICOR) when assessing the BOLD signal for neurofeedback‐induced changes over time (e.g. Sulzer et al., 2013). In a 7 T study investigating the influences of motion, heart rate, heart rate variability, and respiratory volume on amygdala self‐regulation learning effects, Hellrung, Borchardt, et al. (2018) found that neither physiological fluctuations nor motion artefacts were driving factors in learning success. Even so, they did find notable differences in physiological measures between rest and regulation conditions within participants, and recommended the clear reporting of these measures alongside offline physiological noise correction.

## 6 REPORTING PRACTICES REVISITED

Apart from summarising the processing methods used in 128 recent rtfMRI‐NF studies, Figures 6 and 7 in Section 4 highlighted the likelihood that many of the studies' implemented methods remain unreported.

This challenge is not limited to the field of real‐time fMRI neurofeedback and has indeed been described more generally for MRI, including efforts to address it. Nichols et al. (2017) aimed at understanding and improving good practice and reporting standards by creating the COBIDAS guidelines for conducting and reporting all aspects of MRI‐based neuroimaging studies. Related approaches exist in fMRI neurofeedback research, for example, in the form of the TIDieR checklist (Randell, McNamara, Subramanian, Hood, & Linden, 2018) for describing studies in standard terms of ‘diagnostic groups, dose/duration, targeted areas/signals, and psychological strategies and learning models’. The CRED‐nf checklist (Ros et al., 2019) is another laudable example that proposes a standardised checklist that outlines best practices for experimental design and reporting of neurofeedback studies.

Using our improved understanding of real‐time fMRI neurofeedback processing methods from Section 5, as well as building on the above‐mentioned work to improve reporting practices, we have created a COBIDAS‐inspired template to aid researchers in reporting the methods used when calculating their feedback signals. This template checklist should act as a guideline, and we acknowledge that this is not an exhaustive list but one that could mature over time with community input. It was compiled in the vein of the COBIDAS best practice effort and would best be interpreted as an addition to the COBIDAS reporting guidelines for real‐time fMRI. This template is displayed in Table 2, and an online version is available at https://osf.io/kjwhf/.

**TABLE 2:** A COBIDAS‐inspired template for the reporting of processing and quality control steps in real‐time fMRI neurofeedback

Category	Reporting suggestions
General (items apply to all categories)	Report the space in which each real‐time processing is performed (i.e. native volume, native surface, MNI volume, template surface, native structural, other) Report whether real‐time processing steps are executed on the whole brain, within a region of interest (ROI), or on the calculated neurofeedback signal Report the order in which real‐time pre‐processing steps were implemented Provide reasoning if steps were not implemented For custom implementations, specify details
Software (items apply to all categories where software use is reported)	Software used for real‐time processing (e.g. Turbo BrainVoyager, AFNI, SPM + Matlab, OpenNFT, BART, FRIEND, BioImage Suite, Other, Custom) Software used for offline processing (with clear distinction from real‐time processing) Indicate when default settings for the implemented software were used For each software used, be sure to include version number, revision number, URL and Research Resource Identifier For custom software/scripts, provide dependencies and link to code if possible
Slice time correction	YES/NO Name of software/method Whether performed after or before motion correction Reference slice Interpolation type and order (e.g. third order spline or sinc)
Motion correction	YES/NO Name of software/method Use of non‐rigid registration, and if so the type of transformation Use of real‐time motion susceptibility correction (fieldmap based unwarping), as well as the particular software/method Reference scan (e.g. a template volume from the pre‐real‐time scans or the first volume of the real‐time session) Image similarity metric (e.g. normalised correlation, mutual information etc.) Interpolation type (e.g. spline, sinc), and whether image transformations are combined to allow a single interpolation Use of any slice‐to‐volume registration methods, or integrated with slice time correction Explanation of software and hardware used in the case of prospective motion correction
Function–structure (inter‐subjective) co‐registration	YES/NO Name of software/method Type of transformation (rigid, non‐linear); if non‐linear, type of transformation Cost function (e.g. correlation ratio, mutual information, boundary‐based registration etc.) Interpolation method (e.g. spline, linear) Distinguish between coregistration applied pre‐real‐time (e.g. to support real‐time operations like tissue masking) and coregistration done in real‐time
(Gradient) distortion correction	YES/NO Specify if implemented as part of real‐time acquisition sequence on (as opposed to as a real‐time processing step)
Spatial smoothing	YES/NO Name of software/method Size and type of smoothing kernel Filtering approach, for example, fixed kernel or iterative smoothing until fixed FWHM
Nuisance regression	YES/NO Specify software and GLM algorithm type (e.g. cumulative, windowed, incremental) with applicable parameters (e.g. window length) If head motion parameters are included, report the expansion basis and order (e.g. first temporal derivatives; Volterra kernel expansion) If tissue signals are included, report the tissue type (e.g. whole brain, grey matter, white matter, ventricles), the tissue definition (e.g. a priori seed, automatic segmentation, spatial regression) and signal definition (e.g. mean of voxels, first singular vector etc.) Report any other included regressors and how they are calculated
Detrending/drift removal	YES/NO Name of software/method (e.g. nuisance regression using a real‐time GLM with linear and/or cosine basis set regressors; exponential moving average filter; custom filter) If nuisance regression is used, specify the order of regressors and/or cutoff frequency If nuisance regression is used, specify GLM type (cumulative, windowed, incremental) with applicable parameters (e.g. window length)
Physiological noise removal	YES/NO Name of software/method If differential regions of interest are used (e.g. to cancel global effects of respiration) specify ROI definition and how the difference is calculated per time step If respiratory and heart rate information are included in real‐time nuisance regression with a GLM, report the modelling choices (e.g. RETROICOR; cardiac and/or respiratory response functions; partial correlation to compartment signals) and number of computed regressors If RETROICOR‐based nuisance regression is used, specify the software/method for computing the regressors and specify how subject physiology traces are accessed in real‐time Distinguish clearly between real‐time physiological noise correction and offline correction and quality checking
High frequency filtering	YES/NO Name of software/method (e.g. modified low‐pass Kalman filter as implemented in OpenNFT to remove high frequency spikes related to subject physiology)
Volume censoring (a.k.a ‘scrubbing’ or ‘despiking’)	YES/NO Name of software/method Criteria for censoring (e.g. real‐time framewise displacement threshold, DVARS threshold, percentage BOLD change threshold, or standardised voxel intensity threshold) Use of censoring (e.g. temporal censoring regressor in real‐time GLM) or interpolation; if interpolation, method used (e.g. spline, spectral estimation)
Serial correlations	YES/NO Name of software/method (e.g. a first‐order autoregressive model AR(1) as implemented in OpenNFT)
Temporal averaging	YES/NO Name of software/method (e.g. a 5‐point moving windowed average applied to the neurofeedback signal)
Intensity normalisation/scaling	YES/NO Name of software/method Scaling factor description (e.g. z‐score normalisation per voxel using the past *n* volumes; whole‐brain intensity scaling to a mean image intensity of constant *k*; voxel efficiency scaling to avoid undesired noise weighting in direct averaging of all voxels within the neurofeedback ROI; scaling of the neurofeedback signal to prevent sudden changes in visual feedback display) Where applicable, provide equations for the scaling of each time step of the volume/ROI/signal
Real‐time data quality control	YES/NO Name of method (e.g. head motion parameter or physiology trace feedback to subject; real‐time display of quality control measures like tSNR to researcher; adaptive acquisition and processing paradigms) Name of software (e.g. AFNI, FIRMM, rtQC, BART, other, custom) Where applicable, provide equations and code for calculating the displayed or monitored parameters
Offline data quality control	YES/NO Name of method/software to check similarity between real‐time and offline exported versions of the neurofeedback session data Name of method/software to calculate general image quality metrics on neurofeedback session data Report if offline physiological noise correction was applied in the assessment of subject‐specific neurofeedback training effects Report if motion parameters or other physiological signals were used post hoc to test as confounds for differences between neurofeedback training groups, or to test for similarities with the task or other fluctuations

Abbreviations: AR, autoregressive; CSF, cerebrospinal fluid; DVARS, differential variance root mean squared; EMA, exponential moving average filter; EPI, echo‐planar imaging; FWHM, full width half max size of Gaussian smoothing kernel; FSL, software library; GLM, general linear model; GM, grey matter; HMP, head movement parameter; HRV, heart rate variability; ICA, independent component analysis; iGLM, incremental general linear model; MVPA, multivariate pattern analysis; PCA, principal component analysis; RETROICOR, retrospective image‐based correction; ROI, region of interest; RT, real‐time; RVT, respiratory volume per time; SNR, signal‐to‐noise ratio; SPM, software library; TR, repetition time; WM, white matter; Y, yes.

## 7 DISCUSSION, RECOMMENDATIONS AND FUTURE PERSPECTIVE

In this work, our goal was to shed light on the status of data quality challenges, denoising practices and methods reporting in the field of real‐time fMRI neurofeedback. Prior studies in conventional fMRI have shown the implications of not sufficiently removing noise signals or not accounting for confounding effects (e.g. Gitelman et al., 2003; Power et al., 2012; Rangaprakash et al., 2018; Van Dijk et al., 2012). We aimed to investigate this in the domain of real‐time fMRI neurofeedback and present our findings such that researchers can be thoroughly informed about the quality of their neurofeedback signal of interest. The aim is to assist researchers in designing rtfMRI‐NF studies that avoid (as far as possible) sham learning and, subsequently, incorrect inferences, and improves (as much as possible) the methods reproducibility of their work through transparent reporting.

### 7.1 Existing denoising methods: Acquisition and processing

Literature showed that methods development during the past two decades has delivered multiple acquisition and processing methods to the researcher conducting an rtfMRI‐NF study, implemented in the form of custom sequences and tools including Turbo‐BrainVoyager, AFNI, OpenNFT, FRIEND and BART. For acquisition real‐time shimming, spiral‐in/out and multi‐echo EPI (including multi‐band) approaches show promise in reducing susceptibility induced geometric distortion and increasing BOLD sensitivity, and are recommended for future implementation.

From a processing perspective, real‐time denoising pipelines showed high similarity to offline counterparts, although some trade‐offs are made because of the time limitation and the iterative nature of real‐time processing. The effects of inclusion or exclusion of specific denoising steps in the real‐time pipeline on the quality of the neurofeedback signal were found to be unexplored except for a single study (Kopel et al., 2019). Table 1 summarised the available real‐time processing methods and made conservative recommendations based on the available evidence, mostly commenting that methods should be piloted to determine their validity for each specific study. At a minimum, 3D volume realignment, drift removal, and signal scaling could be applied, while time‐point smoothing, frequency filtering, and simple nuisance regression using an iGLM could be considered, provided that these methods are first piloted and their effects understood. Researchers are advised against implementing a real‐time iGLM with too many nuisance regressors to avoid overfitting, regressor collinearity and noisy parameter estimates.

It remains difficult to make further empirically supported recommendations for specific denoising pipelines, apart from such general recommendations that are mostly based on evidence from conventional fMRI. This highlights the need for new methodological studies to quantify denoising step effects and compare pipelines. Collection of peripheral physiological data (e.g. heart rate, respiration rate, eye movements) is always recommended when possible, either to be used for real‐time denoising or otherwise to rule out as confounds during offline analysis.

### 7.2 Quality control in real‐time fMRI neurofeedback

Quality control and best practices in rtfMRI‐NF is markedly unexplored and unreported compared to conventional fMRI, where initiatives like MRIQC (Esteban et al., 2017), QAP (Processed Connectomes Project, 2014), COBIDAS (Nichols et al., 2017) support improved quality control and methods reproducibility. Although some studies report the use of best practices and data quality metrics to assess their neurofeedback signal (Koush et al., 2012; Stoeckel et al., 2014; Sorger et al., 2018; Zilverstand et al., 2017), it is unreported in the majority of the literature. Furthermore, other potential data quality issues like differences between offline and real‐time acquired data, or geometric distortion unaccounted for during acquisition or real‐time processing, could further skew the data, yet they remain unreported. It is our perspective that a concerted effort is necessary to establish a practical set of rtfMRI‐NF quality metrics and methods that allow their calculation, visualisation, comparison and reporting. This could expand on the work proposed by Stoeckel et al. (2014) and Thibault et al. (2018).

### 7.3 Methods reporting and best practice adoption

Figure 6 showed that less than a third of the studies reported implementing slice timing correction, spatial smoothing, regression of HMPs, temporal averaging or filtering, outlier or spike removal, using a differential ROI to account for global effects, and further physiological noise correction. While this in itself is not necessarily indicative of insufficient data quality (recall the general absence of empirical evidence for methods recommendations), this low percentage of studies could still raise concern about the general quality of the real‐time fMRI neurofeedback signal. Furthermore, it does indicate a problem with how methods are typically reported, which is an effective hindrance to methods reproducibility.

Ultimately, we should aim for future studies to have the required methodological rigour that allows delineation of the various mechanisms that could drive neurofeedback effects. This creates the imperative that we report accurately and transparently on acquisition, processing and any other steps taken to remove noise fluctuations from and improve the quality of real‐time fMRI and the neurofeedback signal. As a starting point, studies could include a checklist reporting the implementation of the real‐time processing steps listed in this work, as summarised in Table 2 above. An online version of this COBIDAS‐inspired checklist is available at: https://osf.io/kjwhf/.

### 7.4 Future perspective

Moving towards a scenario where the hypothesised usefulness of rtfMRI‐NF in a clinical environment can be investigated and demonstrated transparently will require studies with reproducible methods and results. In light of this, we echo the recommendations made by Thibault et al. (2018) regarding reproducible science. Where possible, rtfMRI‐NF studies with a clear hypothesis should be pre‐registered or follow a registered report submission process. Additionally, the continued use and development of open source software solutions based on widely used neuroimage processing tools, like OpenNFT (SPM), FRIEND (FSL) or AFNI's real‐time plugin, are recommended together with data sharing on platforms like OpenNeuro (https://openneuro.org/). In this way, both published data and methods can be queried by multiple researchers, paving the way for reproducible methods, results and inferences.

## CONFLICT OF INTEREST

R. L. and M. B. are, respectively, employees of Philips Research and Philips Healthcare in The Netherlands. The other authors have declared that no further competing interests exist.

## AUTHOR CONTRIBUTIONS

Stephan Heunis, Rolf Lamerichs, Svitlana Zinger, Bert Aldenkamp and Marcel Breeuwer conceived the manuscript outline. Stephan Heunis conducted the research and wrote the manuscript. Rolf Lamerichs contributed to real‐time fMRI acquisition sections through discussions and writing. Cesar Caballero‐Gaudes and Jacobus F. A. Jansen contributed extensively during manuscripts revision periods. All authors reviewed and revised the manuscript.

## Supporting information


**Data S1** Supplementary Notes.

## Data Availability

The data, code and supplementary material that support the findings of this study are openly available. All supplementary material accompanying this manuscript can be found at: https://dx.doi.org/10.17605/OSF.IO/2N5P3. The 128 studies forming the basis of analyses in this manuscript are listed at: http://bit.ly/rtfmri‐nf‐zotero‐library. The data and code necessary to reproduce figures in this manuscript can be found at: https://github.com/jsheunis/quality‐and‐denoising‐in‐rtfmri‐nf. This code repository also links to an interactive environment that allows exploration and visualisation of the study data.

## Endnote

1 To prevent ambiguous interpretations of the more general terms “reproducibility,” “replicability,” and “repeatability,” in this work we adhere to the definitions for methods‐, results‐, and inferential reproducibility in the biomedical sciences proposed by Goodman, Fanelli, and Ioannidis (2016).
