# Supplementary material for: Quality and denoising in real‐time functional magnetic resonance imaging neurofeedback: A methods review
Source: Hum Brain Mapp. 2020 Apr 25;41(12):3439–67. doi: 10.1002/hbm.25010 (PMC7375116; doi:10.1002/hbm.25010)
Supplement: Supplementary file 1 — Data S1 Supplementary Notes. [file HBM-41-3439-s001.pdf]

Supplementary Notes

This document provides supplementary notes to the manuscript *"Quality and denoising in real-time fMRI neurofeedback: a methods review"* and its supporting documentation and code.

Initially an excel sheet (termed *"Supplementary Table 2"*) was downloaded from the supplementary data provided by Thibault et al. (2018) [1]. An online version of their dataset is available here. This set of 99 studies was used as the starting point for Section 4 of our manuscript, where we investigated the reported implementation of a set of preprocessing and/or denoising steps (amongst others). We extended the search for recent studies by redoing a Web of Science search across All Databases on 9 April 2019 using the same search terms and selection criteria as provided by Thibault et al. (2018), and found another 29 studies in addition to the original 99.

The full text of each article in the list of studies, including supplementary material, were searched for the following key terms: *averag*, band, cutoff, difference, differential, drift, filter, frequency, heart, high, linear, low, motion, movement, nuisance, outlier, parameter, pass, physiol*, respir*, retroicor, scale, scrub, slice, smooth, spike, trend*. We then coded whether these 128 studies reported the use of the following real-time preprocessing steps and other information:

- Slice timing correction (stc)
- 3D volume realignment (mc)
- Spatial smoothing (ss)
- Drift removal (dr)
- 6 head movement parameter (HMP) regression (hmp)
- Temporal smoothing (ts)
- Frequency filtering (ff)
- Outlier/spike removal (or)
- Differential regions of interest (ROIs) (droi)
- Correction for respiratory effects (resp)
- Software use (software)
- Magnet vendor (vendor)
- Magnetic field strength (magnet)

The following abbreviations were used in the coding procedure (i.e. in the Supplementary Sheet):

- BANDPASS = Band-pass filter
- CORR = Correlation
- DNR = Did Not Report
- EMA = exponential moving average filter
- HIGHPASS = High-pass filter
- iGLM = incremental general linear model
- KALMAN = modified Kalman filter implemented in OpenNFT
- LOWPASS = Low-pass filter

- N = No
- REG = Regression
- RETROICOR = Retrospective Image-based Correction
- RT = Real-time
- ROI DIFF = Differential ROI
- TBV = Turbo-BrainVoyager
- Y = Yes
- 2,3,4,5,6PT = Number of time points used for temporal smoothing
- 4,5,6,7,8,9,12MM = FWHM size of Gaussian smoothing kernel

We classified studies as Did Not Report (DNR) if no mention of the particular method was made in the article or supplementary material, and if we could not confidently infer its use from studying the particular article's content. Some studies reported a processing step but did not provide further detail (e.g. "data were spatially smoothed...", with no smoothing kernel size provided). In such cases we coded the study and particular processing step as "Y".

Some entry types were simplified to allow for easier interpretation. Specifically, for Fig.7G where real-time fMRI neurofeedback software use was indicated, some studies (for example Koush et al., 2013 [2]) were classified as OpenNFT even though they were conducted and published before the published release of OpenNFT (Koush et al., 2017 [3]). Because these earlier studies used software developed by the same authors and containing essentially the same infrastructure and processing steps, they were classified for the purposes of our manuscript as using OpenNFT rather than "Custom Matlab + SPM".

An important point that was further examined during the review process is that there could be discrepancies between the default steps implemented in the particular software tool, the steps implemented based on the researchers' choices, and the steps that were eventually reported. Real-time fMRI software defaults could potentially present an accurate reflection of the unreported literature, if it is true that such default steps and parameters were indeed implemented *and* not reported. On the other hand, in the absence of accurate reporting of methods, we can also not be certain that default values were indeed used. Researchers might have had valid reasons for not implementing a specific step, but might still have failed to report this. Thus, whether DNR or the default value is used, an assumption is made in either direction and these have to be balanced.

To balance these unknowns for the data under consideration, we first distinguish between different software implementations in the set of 128 studies. Many (about 24%, see Figure 7G in the main manuscript) were not done with mature software packages but rather with custom pipelines and scripts. We deem these (often one-off) implementations to be more likely to have included the reported steps than to have unreported defaults. On the other hand, more well-known software packages have been used for multiple studies, including Turbo-BrainVoyager (~56% of studies), AFNI real-time plugin (~11% of studies), OpenNFT (~5.5% of studies), BioImage Suite (~3% of studies), and FRIEND (~3% of studies). In these cases we contacted the software developers and asked for their input on standard preprocessing steps and default

values. The general feedback without exception was that, while some default steps and parameters are made available to the users, it is up to the users (and they are indeed encouraged) to select their own pipeline steps and set their own parameters (in some cases with additional plugin functionality, e.g. slice-timing correction in Turbo-Brain Voyager). The developers cannot take responsibility for the accuracy of the information reported in publications. Even so, some possible default steps and parameters were provided by the developers.

With these distinctions, we recoded the dataset such that studied that used mature and widely used software packages reflected default options where particular steps were not reported, while for custom and one-off scripts/software we took the reported information to be accurate as reported. The recoded dataset is available as an additional Tab Delimited Text file as part of the supplementary material. This dataset was re-analysed to generate a new set of figures given below.

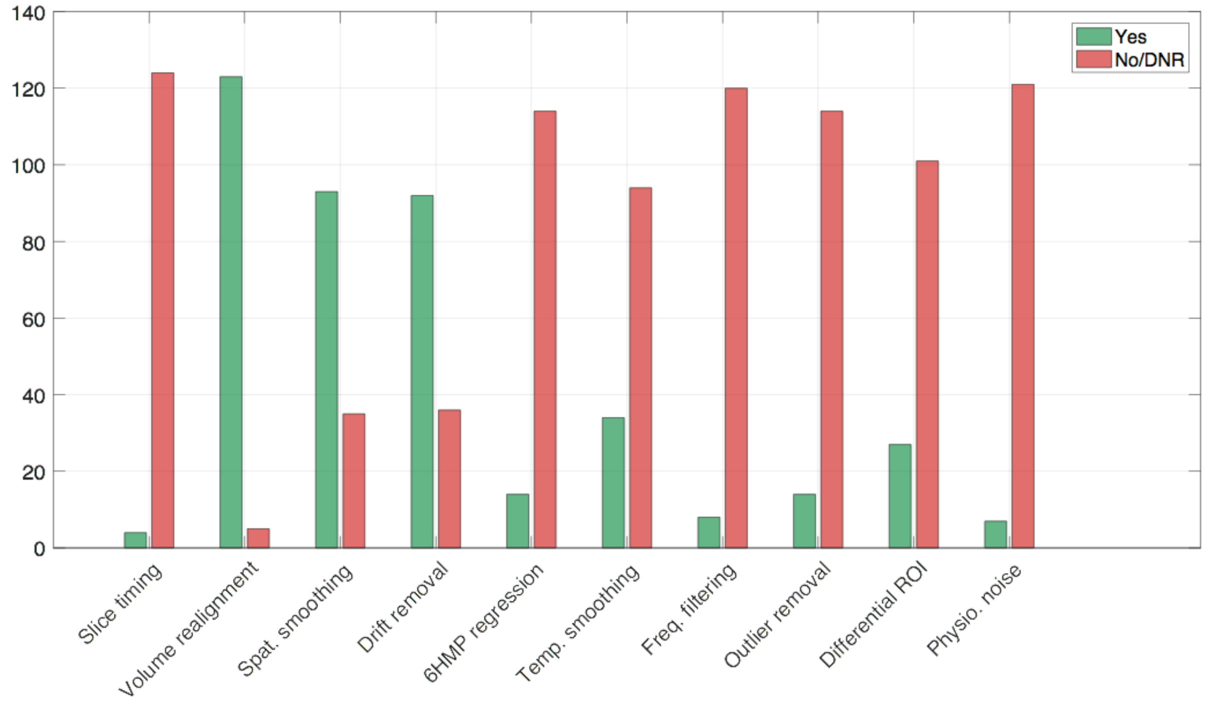

Fig. S1 (above). A list of real-time preprocessing and denoising steps used in 128 recent rtfMRI neurofeedback studies. (DNR = did not report; All bars are indicated as Yes/green and DNR/red while the breakdown for the bar "Differential ROI" is 27 'Yes', 100 'DNR' and 1 'No', Marins et al., 2015).

Fig. S2 (below). Pie charts showing a breakdown of methods used for specific preprocessing and/or denoising steps in the 128 studies compiled in this work, recoded to include default software options. (DNR = did not report; Y = yes, but no further detail reported; N = no):

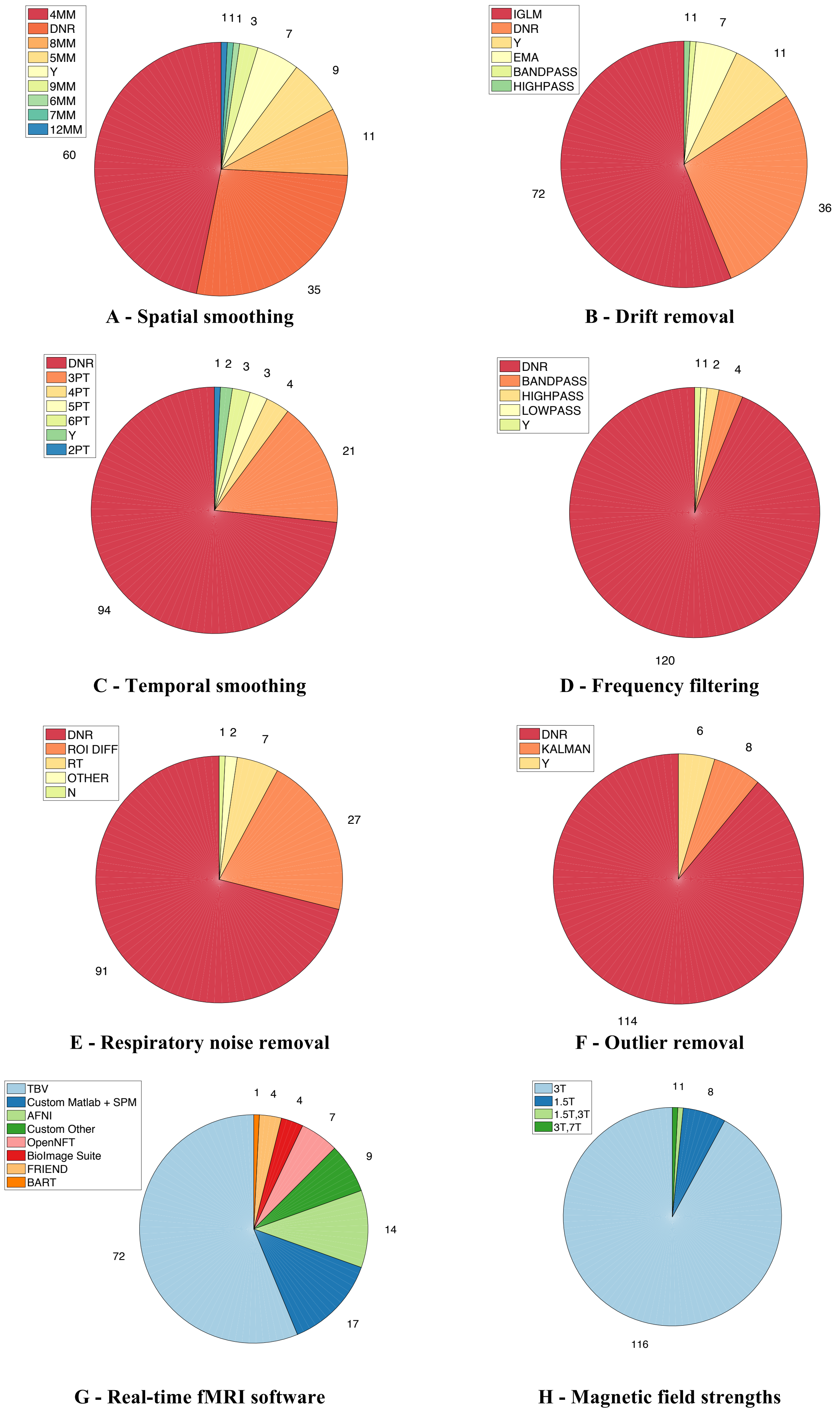
4MM
DNR
8MM
5MM
Y
9MM
6MM
7MM
12MM
1 1 1
3
7
9
11
60
35
A - Spatial smoothing
IGLM
DNR
Y
EMA
BANDPASS
HIGHPASS
1 1
7
11
72
36
B - Drift removal
DNR
3PT
4PT
5PT
6PT
Y
2PT
1 2
3
3
4
21
94
C - Temporal smoothing
DNR
BANDPASS
HIGHPASS
LOWPASS
Y
1 1 2
4
120
D - Frequency filtering
DNR
ROI DIFF
RT
OTHER
N
1 2
7
27
91
E - Respiratory noise removal
DNR
KALMAN
Y
6
8
114
F - Outlier removal
TBV
Custom Matlab + SPM
AFNI
Custom Other
OpenNFT
BioImage Suite
FRIEND
BART
1
4
4
7
9
14
72
17
G - Real-time fMRI software
3T
1.5T
1.5T,3T
3T,7T
1 1
8
116
H - Magnetic field strengths

It is important to note that the above figures, based on recoded information, rely on assumptions made about researchers' reporting methods (as do the figures presented in the main manuscript). Such assumptions and their implications for interpreting the results have been balanced according to what we believe is an accurate reflection of the literature (in the absence of accurately reported processing steps). The reasoning for using or excluding default options, as well as the recoded data and the code to reproduce the results, are presented here to allow the reader to generate and interpret the results in further ways that they see fit.

Data and code availability

The 128 studies are all available as references in a public Zotero group collection: http://bit.ly/rtfmri-nf-zotero-library. This collection, if opened through the Zotero desktop app, can be exported in JSON format and used with our publicly shared scripts (https://github.com/jsheunis/quality-and-denoising-in-rtfmri-nf) to reproduce the manuscript figures. The JSON file and a tab-delimited text file containing the list of coded studies are also available online (https://osf.io/e752r/) and can be used directly with the provided scripts.

References

1. Thibault, R.T., MacPherson, A., Lifshitz, M., Roth, R.R., Raz, A., 2018. Neurofeedback with fMRI: A critical systematic review. NeuroImage 172, 786–807. https://doi.org/10.1016/j.neuroimage.2017.12.071
2. Koush, Y., Ashburner, J., Prilepin, E., Sladky, R., Zeidman, P., Bibikov, S., Scharnowski, F., Nikonorov, A., De Ville, D.V., 2017. OpenNFT: An open-source Python/Matlab framework for real-time fMRI neurofeedback training based on activity, connectivity and multivariate pattern analysis. NeuroImage 156, 489–503. https://doi.org/10.1016/j.neuroimage.2017.06.039
3. Koush, Y., Rosa, M.J., Robineau, F., Heinen, K., W. Rieger, S., Weiskopf, N., Vuilleumier, P., Van De Ville, D., Scharnowski, F., 2013. Connectivity-based neurofeedback: Dynamic causal modeling for real-time fMRI. NeuroImage 81, 422–430. https://doi.org/10.1016/j.neuroimage.2013.05.010
